# New chromosome number and cyto-molecular characterization of the African Baobab (*Adansonia digitata* L.) - “The Tree of Life”

**DOI:** 10.1038/s41598-020-68697-6

**Published:** 2020-08-06

**Authors:** Nurul Islam-Faridi, Hamidou F. Sakhanokho, C. Dana Nelson

**Affiliations:** 1 United States Department of Agriculture, Forest Service, Southern Research Station, Southern Institute of Forest Genetics, Forest Tree Molecular Cytogenetics Laboratory, College Station, TX 77843 USA; 2 United States Department of Agriculture, Agricultural Research Service, Thad Cochran Southern Horticultural Laboratory, 810 Hwy 26W, Poplarville, MS 39470 USA; 3 United States Department of Agriculture, Forest Service, Southern Research Station, Forest Health Research and Education Center, Lexington, KY 40546 USA; 4 United States Department of Agriculture, Forest Service, Southern Research Station, Southern Institute of Forest Genetics, Saucier, MS 39574 USA

**Keywords:** Genetics, Cytogenetics

## Abstract

The African baobab (*Adansonia digitata* L.), also referred to as the “Tree of Life”, is a majestic, long-lived and multipurpose tree of sub-Saharan Africa. Internationally, a growing demand for baobab products in the food, pharmaceutical and cosmetics industries has been observed. Considering this, there is a need for scientific information on the genetics and breeding of *A. digitata*, including cytogenetics, genetic diversity and reproductive biology. The objectives of our cytogenetic research were to determine the genome size, chromosome number, and organization of ribosomal DNA (45S and 5SrDNA) of *A. digitata*. Flow cytometry analysis revealed a 2C-DNA value of 3.8 ± 0.6 pg (1Cx monoploid genome size 919.1 ± 62.9 Mbp). Using our improved chromosome preparation technique, we were able to unequivocally count the chromosomes resulting in 2*n* = 4*x* = 168, a revised chromosome number for *A. digitata*. Fluorescent in situ hybridization (FISH) analysis revealed two massively large variants of 45S rDNA and their corresponding nucleolus organizer regions (NOR). The NOR variants were about two to four times larger than the main body of their respective chromosomes. To our knowledge, this is the first report of this phenomenon in a plant species. Furthermore, we found that FISH analysis using the *Arabidopsis*-type telomere repeat sequence probe clarified and confirmed the new chromosome number and characterized the 45S rDNA structural organization.

## Introduction

The African baobab (*Adansonia digitata* L.) is the largest and best known of the eight *Adansonia* species and reported to be native to mainland Africa^1–3^. It is widespread throughout the hot, drier regions of tropical Africa, extending from northern Tanzania and Namibia to Ethiopia, Sudan, and the northern fringes of the Sahara^1^. *Adansonia digitata* is often referred to by its vernacular name, baobab, which is believed to originate from the Arabic word “buhibab” or “fruit with many seeds”^4,5^. The genus *Adansonia* is named after Michel Adanson who brought baobab seeds to Paris in 1754 and who was the first person to provide a comprehensive description and drawing of the tree after his visit to Senegal^5,6^. Despite having hermaphrodite flowers, *A. digitata* is mainly self-incompatible^7^. This majestic tree, known as the “Tree of Life”, is deciduous, reaching up to 20–25 m in height and 20 m in trunk diameter^1,2,8^ and living for hundreds or even thousands of years^9^. A recent radiocarbon dating^10^ study estimated the age of the Glencoe baobab tree located in the Limpopo Province of South Africa to be about 1,800 years^11^.

The baobab is a multipurpose tree that is extensively used for both its nutritional and medicinal values. Virtually, every part of the baobab tree is useful, and the “Tree of Life” lives up to its name as it is reported to have over 300 purposes including providing nutritious food, livestock fodder, fiber, medicine, and income to local people^12–18^. Edible parts of the tree include leaves, seeds, and fruit pulp, which are a good source of vitamins and minerals^19,20^. The pulp is generally used for juice, snacks, sweet preparations, fermenting agent in brews, porridge and in food recipes^21,22^. Because of its nutritional and health benefits, the baobab fruit pulp has been identified as a suitable candidate as a new source of functional foods, drinks and cosmetics^23^ likely explaining the acceptance of the baobab fruit pulp as a novel food ingredient in the European Union^24^ and the United States^25^. The growing interest for baobab products is raising concern that fruit production for international trade might lead to overexploitation of natural stands^16,17^. In the meantime, natural stands of baobab are already under threat due to climate change and human activities such as land clearing by an ever-increasing population. Genetic information is needed for *A. digitata* to help remediate this threat through the development of informed gene conservation and species domestication strategies.

The genus* Adansonia* L. belongs to Bomacoideae, a subfamily of Malvaceae, and consists of eight species, seven of which are diploids (2*n* = 2*x* = 88) and six of these species (*A. grandidieri* Baill., *A. suarezensis* H. Perrier, *A. rubrostipa* Jum. & H. Perrier, *A. za* Baill., *A. madagascariensis* Bailll., and *A. perrieri* Capuron) are native to Madagascar with the seventh (*A. gregorii* F. Muell.) being native to northwestern Australia. *Adansonia digitata* is the only tetraploid species in the genus and it is native to mainland Africa, although it has been introduced throughout the tropics via humans and trans-ocean currents^12,26–30^. A report of a ninth baobab species, *A. kilima* Pettigrew et al.^30^, native to eastern and southern Africa suggested that it is diploid (2*n* = 88) but has been challenged by Cron et al.^31^ who concluded that *A. kilima* and *A. digitata* are synonyms. Chromosome number, genome size, and ribosomal DNA distribution have been routinely used to study phylogenic relationships in plants and these cytological features have showed importance in evolutionary biology for many species^32–35^.

Several reports on chromosome number of *A. digitata* are available, but these reports differ widely, ranging from 2*n* = 96^36^, 2*n* = 128 (Schroder 1982, personal communication reported by Wickens & Lowe^29^), 2*n* = 144^37–39^, 2*n* = 160^26,35^ to 2*n* = 166^31^. We also found conflicting reports of the genome size of *A. digitata*^31,35,42^, ranging from about 3 to 7.7 pg 2C as measured with Feulgen microdensitometry or flow cytometry. Given these conflicting results for chromosome number and genome size of *A. digitata*, we decided to reevaluate these parameters using updated techniques. For chromosome number we used a root tip protoplast technique for chromosome spreading^40,41^ and for genome size we used flow cytometry. Chromosome banding using Chromamycin A3 (CMA3) involving *A. digitata* has been reported^35^, but no reports on fluorescence in situ hybridization (FISH) using rDNA or other probes in this species are available. Given these conflicting or missing data for *A. digitata*, our objectives in this study were to: (1) provide an accurate estimate of genome size, (2) establish the chromosome number and (3) determine the number, chromosomal location, and structural organization of the ribosomal DNA (45S and 5S rDNA).

## Results

### Flow cytometry

For each sample, flow cytometry analysis provided counts of nuclei to fluorescence intensity values (displayed as histograms), fluorescence intensity means, and coefficients of variation (Fig. S1). The CV values ranged from 1.98 to 4.12 with a mean of 2.53. An illustrative histogram of the relative DNA content with two peaks corresponding to the G1 nuclei of *A. digitata* and *Solanum lycopersicum* L. cv. ‘Stupicke’ is shown in Fig. S1. The mean 2C nuclear DNA content and 1Cx monoploid genome size of *A. digitata* seedlings were 3.8 ± 0.6 pg and 919.1 ± 62.9 Mbp, respectively (Table 1).

**Table 1:** *Adansonia digitata* L. 2C (pg), 1Cx (pg) nuclear DNA content and 1Cx monoploid genome size (Mbp) determined by flow cytometry, and chromosome count determined by cytogenetic analysis.

Variable	Mean ± SD	Minimum	Maximum
2C-DNA (pg)*^a^	3.80 ± 0.6	3.44	4.3
1Cx-DNA (pg)*^a^	0.94 ± 0.1	0.86	1.1
1Cx genome size (Mbp)*	919.1 ± 62.9	841.1	1,055.1
Chromosome count*^b^	167.9 ± 1.12	164	172

*^a^The mean is the average of 20 runs from 10 plants (2 runs/plant).

*^b^The mean is the average of 94 counts (3 counters, 31 or 32 cells per counter).

### Chromosome count

**Figure 1:**
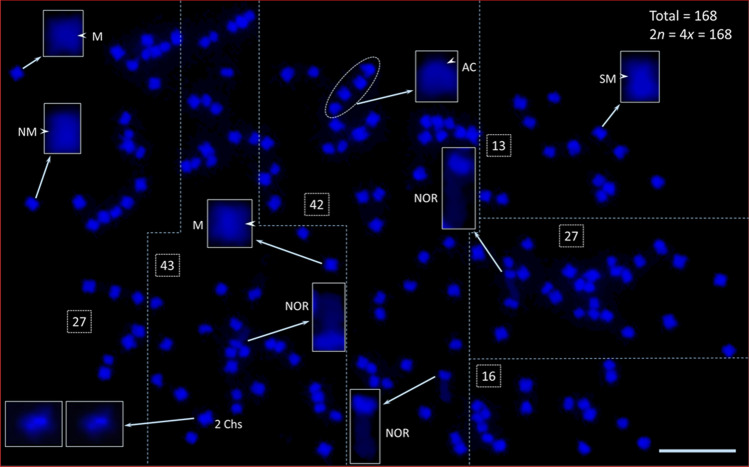
Root tip somatic chromosome spread from African baobab *A. digitata* L. (Seedling-2). The total number of chromosomes is 168 (2*n* = 4*x* = 168), and they range from acrocentric to metacentric. Some of these chromosomes are shown in inserts (enlarged images of individual chromosomes) (*M* metacentric, *NM* near metacentric, *SM* sub-metacentric, *AC* acro-centric; the arrow heads point at the primary constrictions on individual chromosomes). Three massive Nucleolus Organizer Region (NOR) chromosomes were observed in Seedling-2 (see enlarged images marked as “NOR”). One smaller chromosome was masked by a larger one (see lower left-hand side boxes as shown in inserts, so the count should be two). Four chromosomes in oval encircled dotted line (at the top, center, are acro-centric chromosomes). To facilitate counting, the spread was divided into six sections of 27, 43, 42, 13, 27 and 16 chromosomes, respectively, for a total of 2*n* = 4*x* = 168. Scale bar is 5 µm.

We counted chromosomes numbers from 32 intact cells with well separated chromosomes. Three independent counts were made on the same 32 individual cells, providing strong support for 168 chromosomes (Table 1; Fig. 1; Fig. S2). Furthermore, following FISH with *Arabidopsis*-type telomere repeat sequence (ATRS) probe [(TTTAGGG)n], each chromosome arm showed a pair of telomere signals, making it easier to count the total chromosome number in each cell (Fig. 7). These data provide strong evidence that the *A. digitata* chromosome number is 2*n* = 4*x* = 168 (Table 1), and structurally they can be designated as metacentric, near-metacentric, sub-metacentric, near sub-metacentric and acrocentric (Fig. 1).

### 45S rDNA FISH

**Figure 2:**
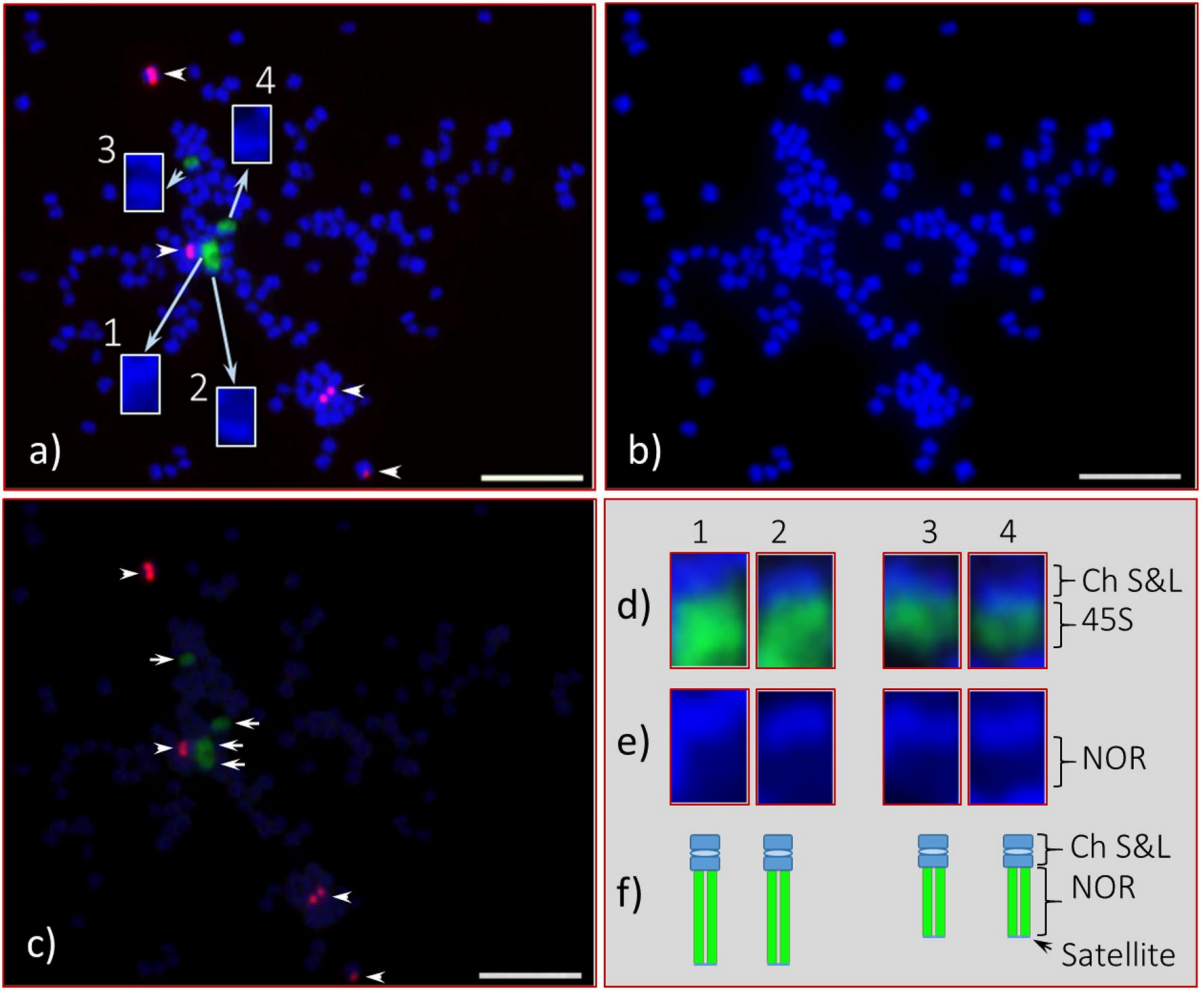
Fluorescent in situ hybridization (FISH) of 45S and 5S rDNA in somatic chromosome spreads of *Adansonia digitata* L. (Seedling-1). (**a**) Four 45S rDNA FISH signals (green) observed at the end of four chromosomes (also shown in “d”, enlarged image of each), and they are marked as 1, 2, 3, and 4 (inserts are enlarged DAPI stained chromosomes). (**b**) DAPI-stained chromosomes of the same cell as in “a”. (**c**) Same cell captured with reduced DAPI exposure that shows bright 45S (green, arrows) and 5S (red, arrowheads) rDNA signals. (**d**) Enlarged image of four 45S rDNA-bearing chromosomes with corresponding DAPI-stained chromosomes in “**e**”, depending on FISH signal intensities 1 and 2 are most likely a homologous pair and same for 3 and 4; (**f**) diagrammatic representation of these two homologous pairs of 45S rDNA bearing chromosomes. Scale bar is 5 µm.

**Figure 3:**
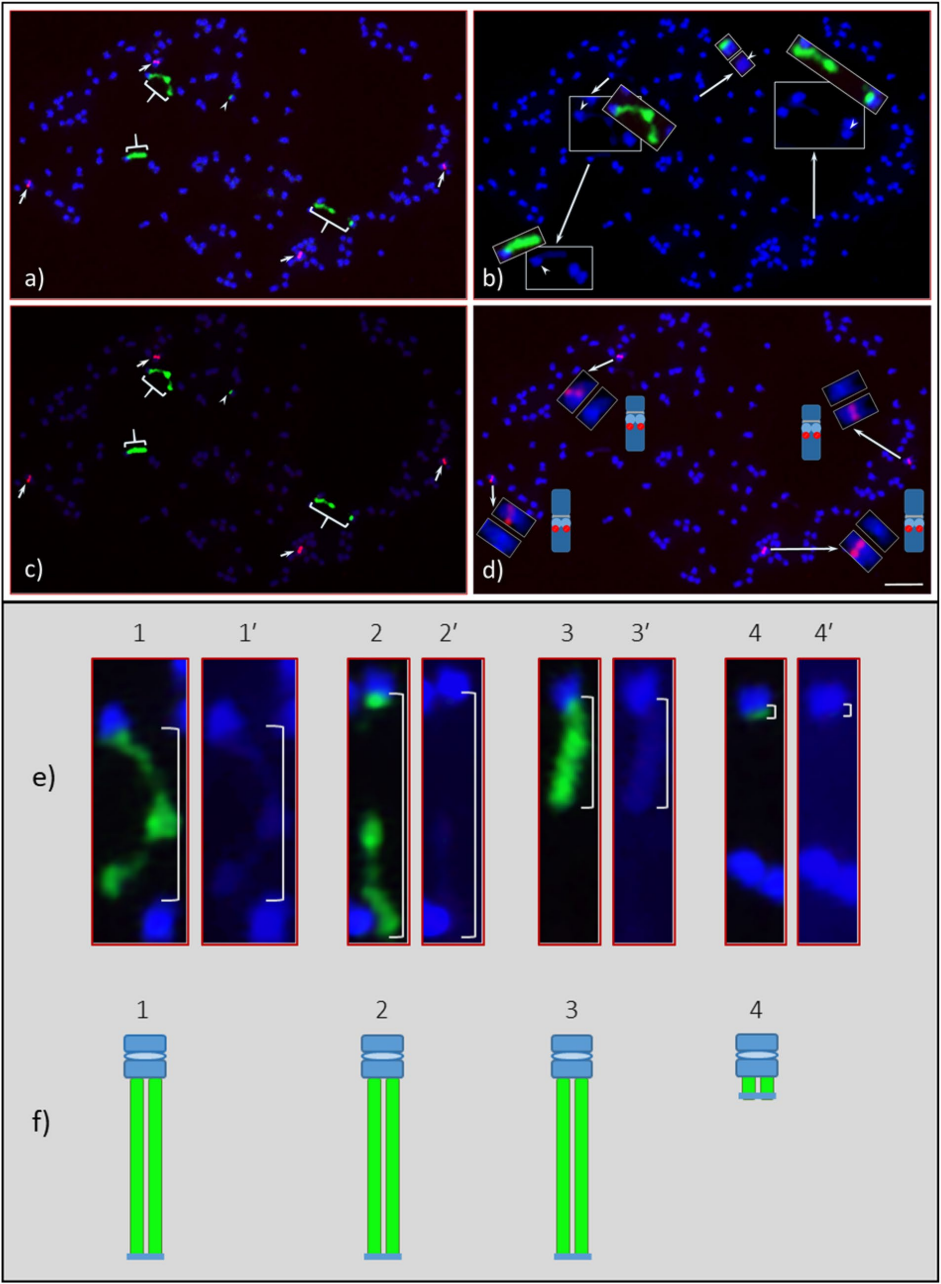
Root tip somatic chromosome spreads of *Adansonia digitata* L. (Seedling-2) analyzed with 45S and 5S rDNA oligonucleotides probes. (**a**) Four 45S rDNA signals (green, three braces and one arrowhead) observed at the end of four chromosomes (also shown in “e”, enlarged image of each and marked as 1, 2, 3, and 4), three of them are as large as in Seedling-1, and the fourth one is very small (arrow head), and four 5S rDNA observed in the middle of four chromosomes (arrows). (**b**) DAPI-stained chromosomes of the same cell as in “a”, but the inserts are images of 45S rDNA-bearing chromosomes along with DAPI images and their respective centromere position marked as arrow heads. (**c**) The same cell captured with reduced DAPI exposure that shows bright 45S (green, three braces and one arrowhead) and 5S (red, arrows) rDNA signals with very light DAPI stained chromosomes in the background. (**d**) Same cell as in “a” but showing 5S rDNA (Cy3, red signals) in DAPI background chromosomes, the inserts showing enlarged images of each of the 5S rDNA bearing chromosomes along with DAPI stained chromosomes and a diagrammatic representation of each. (**e**) Enlarged images of four 45S rDNA bearing chromosomes (1, 2, 3, and 4) along with corresponding DAPI-stained chromosomes (1´, 2´, 3´ and 4´), depending on signal intensities 1 and 2 are most likely a homologous pair and same for 3 and 4. (**f**) Diagrammatic representation of these two homologous pairs of 45S rDNA bearing chromosomes. Scale bar is 5 µm.

Four 45S rDNA signals were observed across the cell spreads for both seedlings studied; however, as revealed by the intensity of the FISH signals, the 45S rDNA copy number is massively large for each of the four chromosomes for Seedling-1 (Fig. 2) and for three of the four chromosomes for Seedling-2 (Fig. 3). For Seedling-2, the 45S rDNA signal observed was much reduced (approximately 90 to 95%) for the fourth chromosome compared to any of the other three showing FISH signals (Fig. 3). This indicates that the 45S rDNA sites or nucleolar organizing regions (NORs) are extremely long; up to approximately four times longer than the main body (both long and short arms) of the metaphase and pro-metaphase chromosomes [(Figs. 1 (inserts labeled as NOR), 2, 3e1-3, f1-3, 4; Fig. S3)]. In addition, a slight curving bulge can be observed in the middle of the main body of some of these chromosomes with 45S rDNA signal [Fig. 3b; Fig. S3h4 (arrowheads)], indicating the potential metacentric status of these chromosomes.

**Figure 4:**
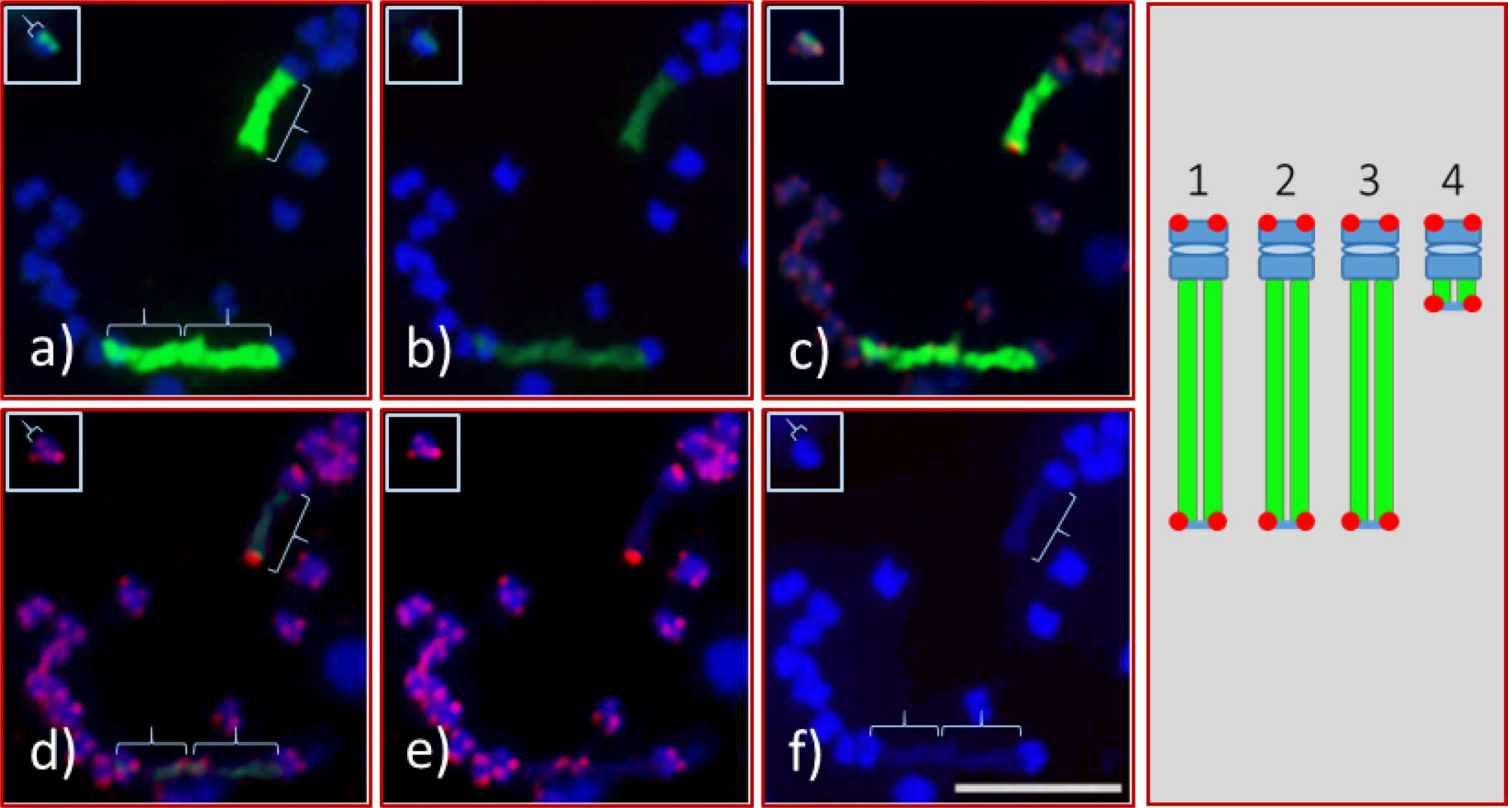
A portion of a metaphase chromosome spread of *Adansonia digitata* L. (Seedling-2), with all three massive 45S rDNA-bearing chromosomes and the fourth chromosome shown in inset (top-left hand side corner). (**a**) An FITC (fluorescein isothiocyanate) image (green signal, curly bracket) of 45S rDNA was captured with automatic camera mode (to obtain a very bright green image). (**b**) The image of same spread as in “a” was captured with bright DAPI and reduced FITC in controlled camera mode to display bright chromosome background with low green (45S rDNA) signal. (**c**) Same spread as in “a” with 45S rDNA and ATRS probe signals. (**d**) Same spread as in “a”, image was captured under reduced FITC that displays faint green string of green signal (curly brackets) where the ATRS signals are brightly visible at the end of the respective NORs. (**e**) Same spread as in “a” without green signal. (**f**) Same spread as in “a” with only bright DAPI-stained chromosomes. The right-hand side Figure is a diagrammatic representation of the four 45S rDNA chromosomes of Seedling-2. Scale bar is 5 µm.

### 5S rDNA FISH

Four 5S rDNA FISH signals [red, Figs. 2a,c (arrowheads), 3a,c,d (arrows), 5b,c,e (arrows), 7a (arrowheads)] were consistently observed in both seedlings across the cell spreads (interphase to metaphase chromosomes). The 5S rDNA sites are located interstitially and proximally (near the centromere positions) in two pairs of homologous chromosomes along with bright DAPI bands, which are co-localized with the 5S rDNA FISH signals on the long arms of both homologues (Fig. 3d). One of the homologous pairs is a sub-metacentric chromosome and the other is near-metacentric. A diagrammatic representation of each of the 5S rDNA bearing chromosomes is shown in Fig. 3d (also see enlarged images in inserts, DAPI stained and FISH signals chromosomes). These enlarged chromosomes clearly show the physical location of the 5S rDNA sites. The signal intensities observed varied for the sub-metacentric pair and could be an indication of 5S rDNA copy number variation^32,43,44^.

**Figure 5:**
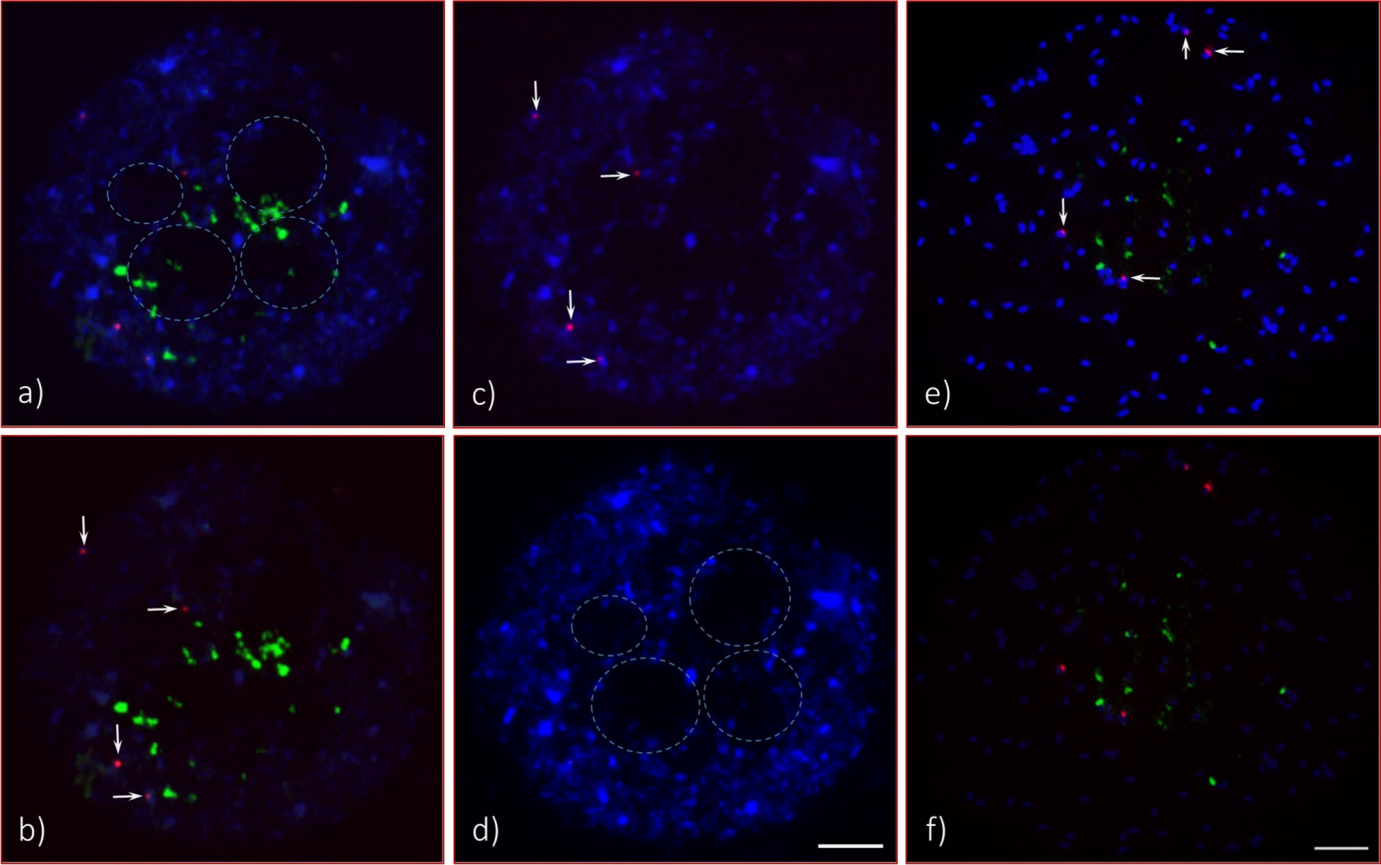
Numerous 45S (green signals) and four 5S (red signals) observed in interphase and prophase cells of *Adansonia digitata* L. (Seedling-2). (**a**) As many as 30 + 45S rDNA FISH signals can be observed. (**b**) Same cell as in “a”, but the image was captured with short exposure time under DAPI filter to show bright green (45S rDNA) and red signals (5S rDNA, arrows). (**c**) Same cell as in “a”, showing 5S rDNA signals (arrows) in DAPI background interphase cell. (**d**) Same cell as in “a”, showing four hallow encircled areas, which are presumptive spaces for four nucleoli. (**e**) Late prophase cell with scattered green signals (45S rDNA) and four 5S rDNA signals (red, arrows) in DAPI background chromosomes. (**f**) Same cell as in “d”, about fifteen green signals (45S rDNA) and four red signals (5S rDNA) in reduced DAPI to show the brightness of the signals. Scale bars are 5 µm.

### Interphase and prophase 45S and 5S rDNA FISH

We observed numerous dispersed 45S rDNA FISH signals in interphase and prophase cells. DNA in interphase nuclei are highly decondensed, so as expected numerous (as many as 50 minor to major) 45S FISH signals are sporadically distributed in the nucleus (green, Fig. 5a,b; Fig. S4a,c, S7a). Depending on the orientation of interphase nuclei, there is a maximum of four hollow areas that can be observed, and these are the potential bodies of nucleoli, which cannot be stained with DAPI because they are mainly composed of RNA. A group of four hollow areas can be clearly observed in Fig. 5a,c,d, and Fig. S4b,d. In prophase cells, the 45S rDNA displayed large blocks of FISH signals in addition to numerous scattered FISH signals (green, Fig. 5e,f; Figs. S5a,c, S6, S7b). Furthermore, various numbers of 45S FISH signals were observed even in early metaphase (Figs. 3a–c, 7) as expected given the partially decondensed state of the DNA at this stage.

### *Arabidopsis*-type telomere repeat sequence (ATRS) FISH

**Figure 6:**
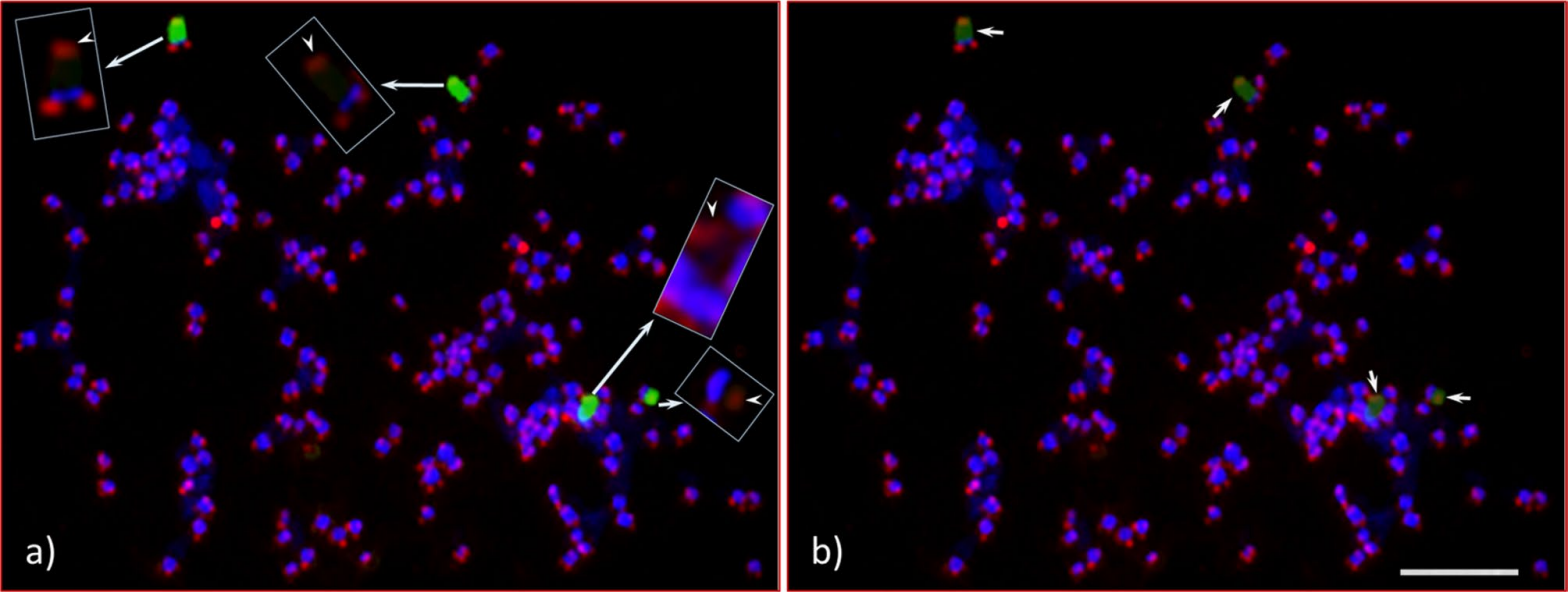
Root tip chromosome spreads of *Adansonia digitata* L. (Seedling-1), analyzed with 45S rDNA and ATRS-type telomere oligonucleotide probes. (**a**) Four 45S rDNA (green) and telomere repeat (red) signals in DAPI-stained chromosomes background, inserts are the enlarged images of 45S rDNA-bearing chromosomes (with no green signals) showing the telomere signals at both ends of each chromosome, arrow heads show the telomere signals at the terminal end of each 45S rDNA signals. (**b**) Same cell as in “a” captured with short exposure time under FITC filter, the telomere signals at the terminal end of each 45S rDNA signals (light green signals) are brightly visible compared to “a”. Scale bar is 5 µm.

**Figure 7:**
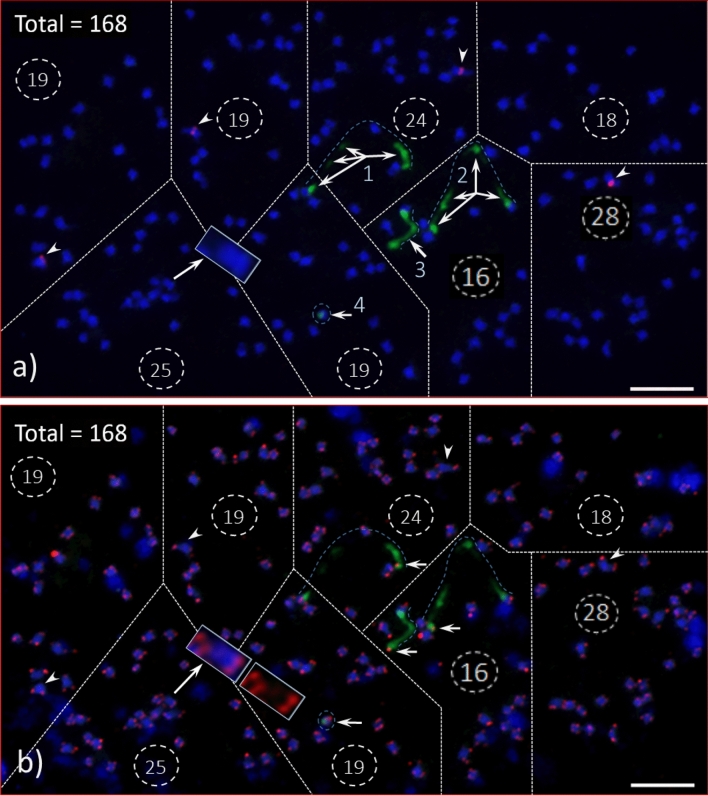
*Adansonia digitata* L. (Seedling-2) pro-metaphase chromosome spread analyzed with 45S and 5S rDNA probes (1st hybridization, “**a**”) and re-hybridized with 45S rDNA (as a control) and ATRS probes (2nd hybridization or re-hybridized, “**b**”) a) As many as thirteen 45S rDNA (green signals, arrows) from four NOR bearing chromosomes (dotted lines over green signals numbered as 1, 2, 3 and 4) and four 5S rDNA (red, arrowheads) signals, the spread is divided into eight sections to facilitate accurate counting of chromosomes and the total number is 168 (19 + 19 + 24 + 18 + 25 + 19 + 16 + 28 = 168). One large metacentric chromosome (see insert in the bottom left-hand side section) may appear as two chromosomes (due to its sharp constriction in the center of the chromosome). In such scenario, (**a**) ATRS hybridization (see insert in “**b**”) would be the option to confirm a single chromosome; (**b**) The same cell was re-hybridized (i.e., 2nd hybridization, see Method for details) with 45S rDNA (as control, green signal) ATRS probes (red signals) after washing off probes from the 1st hybridization. The 5S rDNA signals are missing (arrowheads), which confirmed the 1st hybridization probes were completely washed off. Though the terminal end of each 45S rDNA-bearing chromosomes showed no visible sign of satellites, the telomere signals confirmed (arrows) that these chromosomes are protected by telomere repeat DNA sequences. The same large metacentric chromosome (insert in “**a**”) showed a pair of ATRS signals (red) at each end (inserts in “**b**”) and no signals in the middle of the chromosome that further confirmed as one chromosome, and the total count was 168. Scale bars are 5 µm.

A satellite is a structural body of a chromosome that is typically located at the end of a NOR—the site of major 45S rDNA locus. Since there were no visible signs of a satellite distal of the 45S FISH signals for any of the 45S rDNA-bearing chromosomes [Figs. 2a,e, 3e (1´, 2´ and 3´), 4f)], we used a separate FISH with the ATRS probe^45^ to further investigate the ends of the NORs. As expected, the end of each chromosome arm showed a pair of ATRS signals i.e., one signal on each of the two chromatids (Figs. 4c–e, 6, 7b; Fig. S3). Sometimes one larger signal was observed instead of two smaller ones, which is due to the orientation of a chromosome, i.e., if one chromatid is aligned on top of the other one, polar view, yielding one larger signal (Fig. 6a, arrowheads). Furthermore, due to the decondensed nature of DNA numerous ATRS signals were seen throughout the interphase nuclei (Figs. S7a). Due to the extended exposure time necessary for capturing ATRS signals (red), some of the signals appeared to be very large (Fig. 6). In terms of chromosome counting, sometimes a large, metacentric chromosome can be counted as two due to its sharp primary constriction (see insert, Fig. 7a), but this can be clarified by ATRS FISH as shown in Fig. 7b (see inserts) where each chromosome end showed a pair of signals.

## Discussion

### Flow cytometry

Nuclear DNA content of *A. digitata* has been reported in previous studies^31,35,42^. Bennett and Leitch^42^ reported 1C and 2C values of 3.9 and 7.7 pg, respectively. A Feulgen microdensitometry method was used to obtain these values unlike flow cytometry as used in our study. Using the flow cytometry method with *Zea mays* L. ‘CE-777′ (2C = 5.43 pg) and *Glycine max* Merr. ‘Polanka’ (2C = 2.50 pg) as standards, Costa et al.^35^ reported 1.67 pg/1Cx for *A. digitata*, but they did not provide details of their experimental procedure. On the other hand, Cron et al.^31^ reported 3.04 pg/2C for the nuclear DNA content of *A. digitata*, which is similar to our results. Additionally, sequencing results of *A. digitata* seed suggested a genome size of about 700 Mbp for the tetraploid African baobab^8^ compared to our estimated monoploid genome size of 919 Mbp. The lack of consistency in nuclear DNA content reports of *A. digitata* can be attributed to the difficulty associated with the genome size estimation of several mucilaginous species. Baobab leaves are particularly rich in mucilage, a polysaccharide compound with sticky glue-like texture rendering the sample viscous, attracting isolated nuclei form clumps or clusters causing difficulties in correct estimation^18,49^. In this study, we overcame this common problem with addition of PVP-40 (≥ 10%) to the staining buffer which resulted in improved resolutions of histograms and lower CV values (mean 2.53) for G1 peaks. Our results suggest that the choice and optimization of the staining buffer is very critical for genome size determination of baobab species.

### Chromosome count

The commonly accepted chromosome number for *A. digitata*, the only tetraploid species in *Adansonia*, is 2*n* = 4*x* = 160^26,30,35^. Numerous earlier reports (1960 to 1974) on chromosome numbers of *A. digitata* ranged from 96 to 144^26^. In our study, only chromosome spreads that appeared to be intact, i.e., well-separated and containing all chromosomes of a complete cell, were photomicrographed and then processed (see “Materials and methods”) for chromosome counting. The chromosomes typically spread out well in mid-prophase to early-metaphase where the terminal ends of some chromosomes appeared to be stained lightly with DAPI (Fig. 1). We provided 32 high quality chromosome spreads, independently, to three individuals trained in cytology for chromosome counting. The mean and standard deviation of the number of chromosomes per cell are 167.87 ± 1.12, with 168 being the median and the mode (Table 1). These data provide strong support for the 2*n* = 4*x* = 168 chromosome number for *A. digitata*. The quality of these spreads and the numbers of replications and independent counts of the chromosomes provided a high degree of confidence, notwithstanding the high chromosome number.

In an earlier study, it was reported that the primary constrictions (the centromere positions) of *A. digitata* chromosomes were not clearly visible^26^. A more recent karyotypic analysis in *Ceiba* species, which, like *A. digitata*, belong to Bombacoideae (Malvaceae), reported that the chromosomes were mostly metacentric^50^. In contrast to *Ceiba* species, we observed different chromosome types such as from metacentrics to acrocentrics (Fig. 1) in *A. digitata*, but not every centromeric position was visible or distinguishable as reported by Baum and Oginuma^26^. Furthermore, we identified that both 45S DNA bearing chromosomes are metacentric or near metacentric (Fig. 3b,e; Fig. S3e) and one of the two 5S rDNA chromosomes is near metacentric and the other sub-metacentric (Fig. 3d).

It is often difficult to obtain well-separated chromosome spreads with full chromosome complements, to accurately determine a species’ chromosome number (and/or ploidy), especially from plants with a high number of small chromosomes (~ 50 and higher). Our modified enzymatic digestion of protoplast technique^40^ works well to prepare chromosome spreads from plant species with a higher number of small chromosomes like *A. digitata* (this report) or *Hibiscus hamabo*^41^. A key feature of this method is allowing the chromosomes to spread naturally on the glass slides without squashing them with cover slips (Figs. 1, 2, 3, 4, 6, 7; Figs. S2, S3).

### 45S rDNA FISH

Recently, 21 different species of the Bombacoideae, including *A. digitata*, were analyzed for CMA3 banding, and nine and ten species excluding *A. digitata* for 45S rDNA and 5S rDNA, respectively, with FISH^35^. It is commonly accepted that the 45S rDNA loci in different species are associated with CMA3 banding^51–53^. *Adansonia digitata* of South American origin was reported to have four-terminal CMA3 bands and occupied the entire half of each chromosome as shown in Costa et al.^35^, but 45S rDNA was not specifically addressed in their report. Our results in *A. digitata* indicate that the structural organization of the 45S rDNA bearing chromosomes are quite different than that of the *A. digitata* studied by Costa et al.^35^. In our study, the main chromosome body of *A. digitata* was about half or less of the size of the NOR when condensed at its maximum, or in other words, the NORs are two to four times larger (depending on the degree of condensation,) than the main body of each 45S rDNA bearing chromosome (Figs. 1, 2, 3, 4, 6, 7; Fig. S3). To our knowledge these are the largest NORs relative to chromosome size observed to date in any plant species.

**Figure 8:**
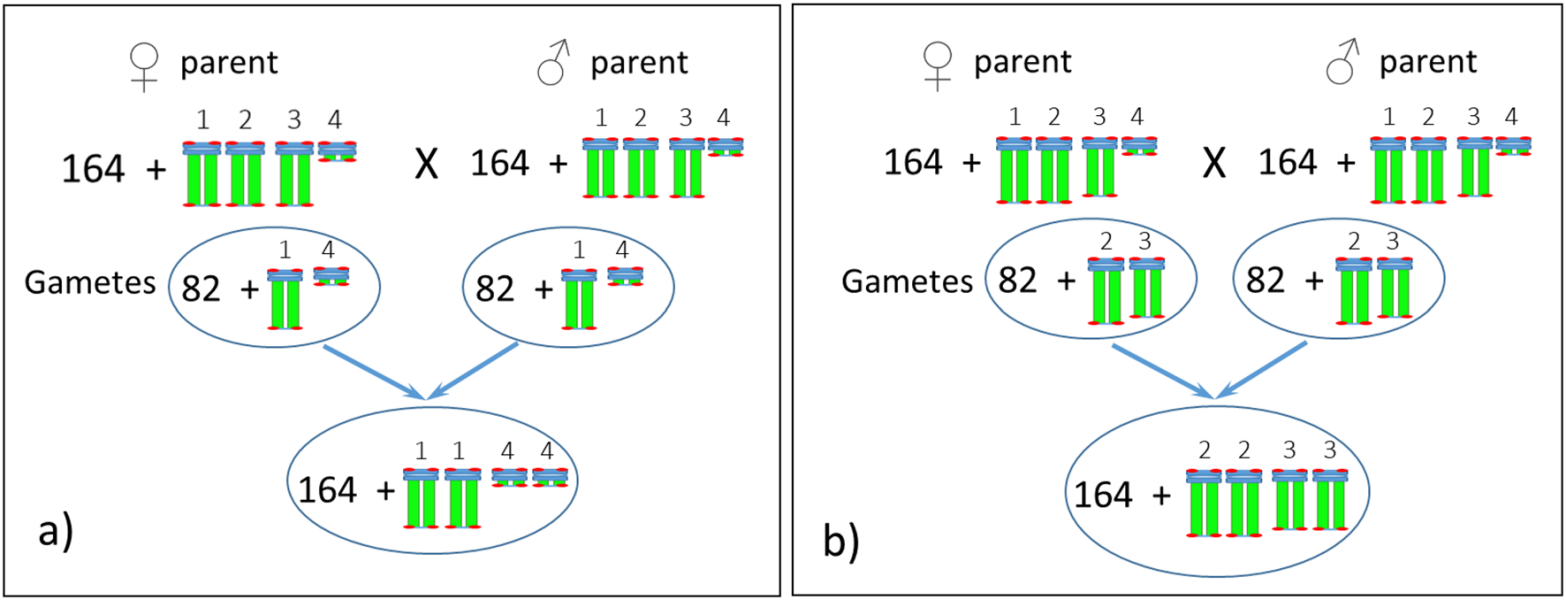
(**a**) Diagrammatic representation of an evolving new sister line of *Adansonia digitata* L. with one complete large nucleolus organizer (NOR) chromosome pair and the second pair with reduced NOR chromosomes. (**b**) This offspring would be same as Seedling-1, and about 50% its offspring would be parental (not shown here).

The organization of the ribosomal DNA is often straightforward to determine; however, it can be more complicated, for example, when the copy number varies between homologues. In the case of *A. digitata* we observed two variants of the 45S rDNA*.* For Seedling-1, both homologous pairs were found to be homozygous with a slight variation in FISH signal intensity (Figs. 2, 6), while for Seedling-2, one homologous pair is homozygous, and the other pair is heterozygous (Figs. 3, 4, 6, 7; Fig. S3). For the heterozygous locus, most of the secondary constriction (i.e., the NOR) of one of the homologues is missing. Loss and gain of rDNA loci and variation of repeat unit number reported in other plant species is a common feature of genome evolution and speciation^44,53–58^. Our results show structural polymorphism in the 45S rDNA loci of *A. digitata*, consistent with ongoing genome evolution, but with only two seedlings analyzed we cannot suggest what the cause may be. The results of Costa et al.^35^ showed that the NORs in their samples (i.e., a tree of South American origin) were similar to those of Seedling-1. This is consistent with the hypothesis that continental Africa was the origin of *A. digitata*^59,60^, with 45S rDNA representative of Seedling-1 being the ancestral type. In any case, the heterozygous variant may eventually become fixed through the evolutionary process, i.e., one pair with the massive 45S rDNA sites and the other pair with a much reduced 45S rDNA locus (see Fig. 8, a diagrammatic representation). Further studies are needed to verify this hypothesis.

### 5S rDNA FISH

For each of the Bombacoideae species reported to date, one 5S rDNA locus has been observed, and with no chromosomal locations specified for *A. digitata*^35^. The diagrammatic sketch provided by Costa et al.^35^ shows metacentric chromosomes containing 45S and 5S rDNA in the 10 Bombacoideae species, with the 5S rDNA locus being placed interstitially towards the middle of the short arm for each species. In contrast, we observed two 5S rDNA loci in *A. digitata*, each located proximally (near the centromeric positions and co-localized with bright DAPI bands) on the long arms of two different chromosomes (Fig. 3d, see inserts, enlarged image of each). One of the 5S rDNA sites was on a near-metacentric chromosome pair and the other on a sub-metacentric pair. This result indicates that *A. digitata* could be an allotetraploid species since two different homologous chromosome pairs carry the 5S rDNA loci, although previous reports suggest it is an autotetraploid^27,30^. Meiocyte analysis of chromosome pairing or genomic in situ hybridization (GISH) should shed light on whether the species is an autotetraploid or allotetraploid or a diploidized tetraploid.

### Interphase and prophase 45S and 5S rDNA FISH

In our study, numerous 45S rDNA FISH signals were observed throughout interphase nuclei and prophase chromosome spreads. Sometimes more than 35 and 50 FISH signals with various intensities (minor to major) were counted in prophase (Fig. S6) and interphase (Fig. S7a), respectively. The interphase nuclei DNA is highly decondensed compared to prophase and metaphase stages indicating the variations of signal numbers are due to the highly decondensed nature of DNA in interphase and, to a lesser degree, in prophase. No prophase, even in late stage, showed four 45S rDNA FISH signals, and this was true for pro-metaphase and early-metaphase stages [Fig. 3, (~ nine 45S FISH signals), 7 (13 45S FISH signals, arrows)]. Data from interphase nuclei provide an upwardly biased count of the number of tandemly repetitive DNA loci such as 45S rDNA. It has been reported that the interphase nuclei cannot be used to determine the 45S rDNA loci number due to their decondensed nature^41,43,48,61^ and this is supported by genome sequencing where rDNA loci are often missing from genome assemblies as they cannot be correctly assembled^47^. We suggest this is also the case for prophase and pro-metaphase when the 45S rDNA is massively large like in *A. digitata*. As the cell progresses from interphase to metaphase, the 45S rDNA FISH signals get markedly denser, so the number of signals gets smaller, and as the chromatin condensation process reaches its maximum level at metaphase, it yields a single strong signal [Figs. 2, 4, 6; Figs. S3, S4 (see model)]. In contrast, the number of 5S rDNA FISH signals remains consistent throughout the cell cycle, from interphase to metaphase. In addition, the 5S rDNA signal intensity is significantly reduced compared to the 45S signals, because the 5S locus contains many fewer copies of rRNA genes compared to the 45S loci.

### *Arabidopsis*-type telomere repeat sequence (ATRS) FISH

Previous work in angiosperms, including forest trees, has shown that the ATRS are confined to the terminal ends (telomeres) of chromosome arms^46–48^.They are composed of five to eight nucleotides and function to protect the chromosome structures from degradation, i.e., maintain the structural integrity of chromosomes^46,62,63^. The 45S rDNA loci (NORs) appeared to be located at the end of four chromosomes (two homologous pairs of chromosomes) in *A. digitata*, without a distal DAPI-stained satellite region, thus hindering the investigation of the chromosome regions distal of the NORs. To explore these regions, we used FISH with the ATRS probe to localize the telomeres relative to the NORs^48,64–67^. The ATRS FISH signals (red) were observed at the distal end of each 45S rDNA signal (green) confirming the presence of telomere and the lack of a typical DAPI-stained satellite (Figs. 4c–e, 6, 7b; Fig. S3). Furthermore, since every chromosome end is protected by telomeres, the ATRS-FISH signals should provide an accurate chromosome count. However, the FISH signals tended to be overexposed when captured using the automated (default) camera mode for TexRd-X (far red fluorochrome) labeled ATRS probe, thus making it difficult to count the chromosomes using the ATRS-signals (red, see Fig. 6). Therefore, to confirm our chromosome counts, we took a different approach, which was to re-FISH the same slides with 45S rDNA as a control (labeled with AlexaFlour 488) and ATRS probe (labeled with Cy3) after washing off the first FISH probes (see “Materials and Methods”). The lack of Cy3 signals (red) from the 5S rDNA sites [(Fig. 7b (arrowheads); S3d (yellow arrowheads)] clearly demonstrates that the probes from the first FISH were completely washed off. The chromosome spread in Fig. 7 was partitioned into eight sections. (19 + 19 + 24 + 18 + 25 + 19 + 16 + 28 = 168) to facilitate chromosome counting (also see Fig. S2). The count perfectly matched the first FISH and the second FISH, providing further support of our chromosome number of 2*n* = 168 (2*n* = 4*x* = 168) for *A. digitata*.

## Conclusion

In this study, we determined the nuclear DNA content, chromosome number, and the distribution and organization of ribosomal DNA (45S and 5S rDNA) of the African baobab tree (*Adansonia digitata* L.). This is the first report of rDNA characterization for the species while 2*n* = 2*x* = 168 constitutes a newly revised chromosome number. Furthermore, our FISH analysis with *Arabidopsis*-type telomere repeat sequence probe clearly demonstrated that the NOR (major 45S rDNA loci) termini are protected by telomere repeat DNAs, and the telomere FISH signals conclusively demonstrated that the chromosome number of *A. digitata* is 2*n* = 4*x* = 168. The cytogenetic methods and cyto-molecular data presented here can serve as the basis for future in-depth investigations in other *Adansonia* species to elucidate their genome structure and evolution as well as for other plant species with a high number of chromosomes. In addition, these findings can be beneficial in developing genetic improvement and conservation strategies for *A. digitata*. Determining the chromosome pairing behavior and utilizing total genomic DNA from diploid *Adansonia* species as labeled probes in genomic in situ hybridization may provide additional information as to the nature of the ploidy of *A. digitata*, revealing whether the species is an autotetraploid or allotetraploid or a diploidized tetraploid.

## Materials and Methods

### Plant materials

Seeds of *A. digitata*, collected in northeastern Senegal, were acid-scarified by soaking them in 98% sulfuric acid (H_2_SO_4_) for 24 h, rinsed thoroughly with tap water in a sink under a fume hood, and sown in soil-containing pots (for flow cytometry) or potting media (for cytology) in a greenhouse. Leaves and root tips were collected from fully developed seedlings and used for flow cytometry analysis and cytology investigation.

### Flow cytometry

Flow cytometry was performed as described by Sakhanokho et al.^41^ with minor modifications to the nuclei staining solution and procedure. In this study, we stained *A. digitata* nuclei with propidium iodide (PI) for analysis with a BD Accuri C6 flow cytometer and a BD Accuri C6 software version 1.0.264.21 (BD BioSciences, Ann Arbor, MI). The staining kit was used following the manufacturer’s instructions. The staining recipe, which was prepared on the day of the flow cytometry procedure, consisted of 20 mL of staining buffer per sample mixed with 120 µL of PI solution, 60 µL of RNAse solution (05-5022; Sysmex Partec GmbH, Görlitz, Germany) and 10% polyvinylpyrrolidone-40,000 (PVP-40, Sigma-Aldrich, St. Louis, Missouri, USA).

Fresh leaves of *A. digitata* seedlings and the internal standard *Solanum lycopersicum* L. cv. ‘Stupicke’ (2C 1.96 pg)^70^ were co-chopped for 30–60 s using 102 mm razor blades (Electron Microscopy Sciences, Hatfield, PA, USA) to equal size (~ 0.5 cm^2^) and placed in a Petri dish before addition of 0.5 mL nuclei extraction buffer. The extraction mixture was filtered through 50 μm nylon-mesh filters (Sysmex America, Inc., Lincolnshire, IL, USA) before addition of 2 mL of staining solution (see above).

The mixture was covered with aluminium foil to protect against light and incubated in a refrigerator at 4 °C for 15 min before being submitted to flow cytometry analysis. We used 10 *A. digitata* seedlings and two leaf samples (technical replicates) per seedling for flow cytometry analysis. A minimum of 5,000 events (nuclei) were gated for each run. Fluorescence ratios, calculated relative to the internal standard reference *Solanum lycopersicum* L. cv. ‘Stupicke’, were converted to DNA content values and expressed in picograms following the formula: Sample 2C-value (picograms) = reference 2C-value × [(Sample 2C mean peak)/(Reference 2C mean peak)]. Genome sizes were converted to megabases (Mbp) using the formula 1 pg = 978 Mbp^68^. Sample monoploid 1Cx-value (pg) was calculated by dividing the 2C-value by the ploidy level of the tetraploid (*x* = 4) *A. digitata*^71^.

### Chromosome spread preparation

Two seedlings (Fig. S8, Seedling-1 and Seedling-2) were used for root tip chromosome spreads and processed separately. Chromosome spreads were prepared following procedures previously described^40,41^ with some modifications. Actively growing root tips about 1.0 cm long were harvested and immediately pre-treated with 2 mM (0.036% (w/v)) 8-hydroxyquilonine for 4.0 h in the dark at room temperature (RT, 22–24 °C), rinsed with ddH_2_O and then fixed in 4:1 (95% ethanol (EtOH): glacial acetic acid (GAA)) and stored at RT over-night before processing for enzyme digestion for chromosome spread. The root tips were processed for enzyme treatment within a week after harvest. Fixed root tips were rinsed with deionized water (di-H_2_O ) to remove the fixative for 30 min at RT, mildly hydrolyzed (0.2 N HCl at 60 °C for 10 min and then 10 min at RT), and rinsed with di-H_2_O (20 min at RT) followed by cold (4 °C) 0.01 M citrate buffer (20 min standing at RT) before enzyme digestion. The enzyme mixture consisted of (2% cellulase RS (w/v, Yakult Pharmaceutical Ind. Co., LTD, Japan), 1% macerozyme R10 (w/v, Yakult Pharmaceutical Ind. Co., LTD, Japan), 2% pectolyase Y23 (w/v, Kyowa Chemical Products, Co., LTD, Japan), 30% cellulase (v/v, C2730, Sigma, USA), 30% pectinase (v/v, P2611, Sigma, USA)] and 40% 0.01 M Citrate buffer (pH 4.5). The enzyme digestion time varied from 24 to 35 min based on the thickness of root-tips. After chromosome preparation, slides containing good spreads were selected and used for FISH within a week or two, or stored at − 80 °C for future use.

### Fluorescence in situ hybridization (FISH) with oligonucleotide probes

We used 45S rDNA pre-labeled oligonucleotide probes (PLOPs) consisting of four each of 18S and 5.8 rDNA 5′-labeled with AlexaFluor 488, three PLOPs of 5S rDNA 5′-labelled with Cy3 as probes cocktail, and one oligonucleotide probe of *Arabidopsis*-type telomere repeat sequence [ATRS; (TTTAGGG)_n_] 5′-labelled with TexRd-X and Cy3 (Table 2; for details see Waminal et al.^45^ to detect the respective rDNAs and telomeres physical locations on *A. digitata* chromosomes. The oligonucleotides sequences were synthesized by Integrated DNA Technologies, Inc. (Coralville, IA, USA).

**Table 2:** List of oligonucleotide probes used for fluorescent in situ hybridization*^a^.

No	Probe	Sequence	Length	Modification
1	18S_UniOP_1	CCGGAGAGGGAGCCTGAGAAACGGCTAC	28	5′AlexaFluor488
2	18SrDNA_UniOP_2	ATCCAAGGAAGGCAGCAGGCGCGCAA	26	5′AlexaFluor488
3	18SrDNA_UniOP_3	GGGCAAGTCTGGTGCCAGCAGCCGCGGT	28	5′AlexaFluor488
4	18SrDNA_UniOP_4	TCGAAGACGATYAGATACCGTCSTAGT	27	5′AlexaFluor488
6	5.8SrDNA_UniOP_1	AAYGACTCTCGGCAACGGATATCTMG	26	5′AlexaFluor488
7	5.8SrDNA_UniOP_2	CWYGCATCGATGAAGAACGTAGCRA	25	5′AlexaFluor488
8	5.8SrDNA_UniOP_3	GCGATACTTGGTGTGAATTGCAGAATC	27	5′AlexaFluor488
9	5.8SrDNA_UniOP_4	GTGAACCATCGAGTYTTTGAACGCAAGT	28	5′AlexaFluor488
5	5SrDNA_ang_1	GGATGCGATCATACCAGCACTAAAGCACCG	30	5′Cy3
6	5SrDNA_ang_2	CCCATCAGAACTCCGAAGTTAAGCGTGCT	29	5′Cy3
7	5SrDNA_ang_3	GCGAGAGTAGTACTAGGATGGGTG	24	5′Cy3
8*^b^	5SrDNA	TCAGAACTCCGAAGTTAAGCGTGCTTGGGCGAGAGTAGTAC	41	5′Cy3
9	Telomere	TTTAGGGTTTAGGGTTTAGGGTTTAGGGT	29	5′TexRd-X, 5′Cy3

*^a^For details see Waminal et al.^45^^.^

*^b^For details see Luo et al.^69^^.^

Freshly prepared slides (3–10 days stored at RT) were treated with RNase-A (30 µg/mL) in 2 × SSC in a coupling jar in a water bath at 37 °C for 60 min followed by two washes in 2 × SSC at 37 °C, 5 min each. The slides were then dehydrated through an ethanol series, 5 min each (70%, 85%, 95% and 100%) at RT and air-dried overnight. The hybridization mixture (25 µL/slide) consisted of 50% deionized formamide (12.5 µL of di-formamide), 10% dextran sulfate (5 µL of 50% ds), 5.0 µg of *E. coli* DNA (used as blocking DNA), 25 ng of each of 45S rDNA (total 100 ng/slide) and 5S rDNA (total 100 ng/slide) oligo probes in 2 × SSC (2.5 µL of 20 × SSC) and adjusted the volume to 25 µL with TE buffer. For telomere site detection, we used 45S rDNA (total 100 ng/slide) as a control along with the ATRS oligo probes (total 100 ng/slide). The hybridization mixture was placed on the spread and covered with a glass-cover slip (22 × 30 mm) without sealing. The slides were then placed in a pre-heated (at 80 °C convection oven) humidity chamber, and then the chromosomal DNA was denatured at 80 °C for 4 min. After denaturation, the slides were cooled down for 2 to 3 min at RT and then placed at 37 °C for incubation for 2 h. After incubation, the glass-coverslip was slid off using a 2 × SSC squeeze bottle, and the slides immediately washed in 2 × SSC at RT for 5 min, two washes in 0.1 × SSC at 40 °C 5 min each, and then washed in 2 × SSC at RT for 5 min followed by quick rinsed in di-H_2_O^41^.

The slides were dried with forced air using a benchtop vacuum pump (GE Commercial Motor, G608GX, Sold by Fisher Scientific INC., USA), the chromosome spreads counter-stained by adding a small drop (10 µL) of Vectashield containing DAPI (Vector Laboratories, USA) and then the preparation covered with a glass-cover slip (24 × 50 mm) to prevent photo-bleaching of the fluorochromes and over-flowing of immersion oil when viewing under the required magnification. For the second FISH, the slides were immersed in 2 × SSC at RT for 20–30 min to slide off the glass-cover slip, washed in 2 × SSC for 1 h and two times in 0.5 × SSC, 30 min each, to remove the probes from the first hybridization. The slides were then dehydrated through an ethanol series as described above and air-dried overnight before performing the second FISH.

### Digital image capture and processing

We used a 63X plan-apochromat oil-immersion objective to view the FISH images with an epi-fluorescence microscope (AxioImager M2, Carl Zeiss Inc., Germany) fitted with suitable filter sets (Chroma Technology, Bellows Falls, VT, USA). Images were captured with a Cool Cube 1 (MetaSystems Group Inc., Boston, MA, USA) high performance charge-coupled device (CCD) camera. Captured images were pre-processed with ISIS v5.1 (MetaSystems Group Inc.) and then further processed with Adobe Photoshop CC 2019 (Adobe Systems Inc., New York, NY, USA) after increasing the resolution from 72 dpi (as photomicrographed) to 300 dpi.

### USDA disclaimer

The use of trade, firm, or corporation names in this publication (or page) is for the information and convenience of the reader. Such use does not constitute an official endorsement or approval by the United States Department of Agriculture, the Forest Service or the Agricultural Research Service of any product or service to the exclusion of others that may be suitable.

## Data availability

All data generated or analyzed for this study are included in this published article as well as its Supplementary Information files.

## Supplementary information

Supplementary information
