# Supplementary material for: New chromosome number and cyto-molecular characterization of the African Baobab (Adansonia digitata L.) - “The Tree of Life”
Source: Sci Rep. 2020 Aug 6;10:13174. doi: 10.1038/s41598-020-68697-6 (PMC7413363; doi:10.1038/s41598-020-68697-6)
Supplement: Supplementary file 1 — Supplementary information [file 41598_2020_68697_MOESM1_ESM.docx]

**Supplementary information**

**New Chromosome Number and Cyto-molecular Characterization of the African Baobab (*Adansonia digitata* L.) – ″The Tree of Life″**

Nurul Islam-Faridi*^1^, Hamidou F. Sakhanokho*^2^, C. Dana Nelson^3,4^

^1^ United States Department of Agriculture, Forest Service, Southern Research Station, Southern Institute of Forest Genetics, Forest Tree Molecular Cytogenetics Laboratory, College Station, TX 77843: ^2^ United States Department of Agriculture, Agricultural Research Service, Thad Cochran Southern Horticultural Laboratory, 810 Hwy 26W, Poplarville, MS, USA 39470; ^3^ United States Department of Agriculture, Forest Service, Southern Research Station, Forest Health Research and Education Center, Lexington, KY, 40546; ^4^ United States Department of Agriculture, Forest Service, Southern Research Station, Southern Institute of Forest Genetics, Saucier, MS 39574

**Fig. S1. Flow Cytometry Result for *Adansonia digitata***

**
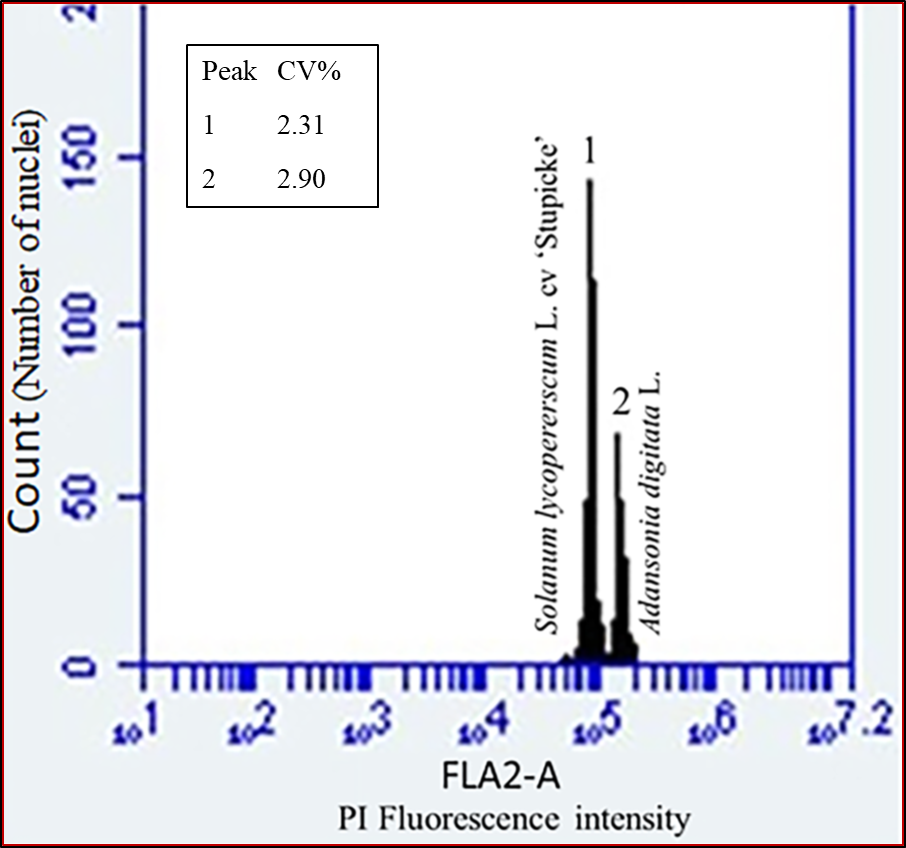
**

**Figure S1.**Representative histogram of nuclear DNA content estimation of *Adansonia digitata L.*

using flow cytometry. Simultaneous analysis of nuclei isolated *Solanum* *lycopersicum* L. cv.

‘Stupicke’ (Peak 1, 2C-DNA 1.96 pg) and *Adansonia digitata* (2*n* = 4*x* = 168), (Peak 2, 2C-DNA

3.80 + 0.6 pg). The two peaks represent populations of nuclei in G1 phase of cell cycle.  The CV values (%) for Peak 1 and Peak 2 were 2.31 and 2.90, respectively**.**

**Fig. S2a. DAPI Stained Chromosome Spread for Chromosome Counting in *Adansonia digitata***


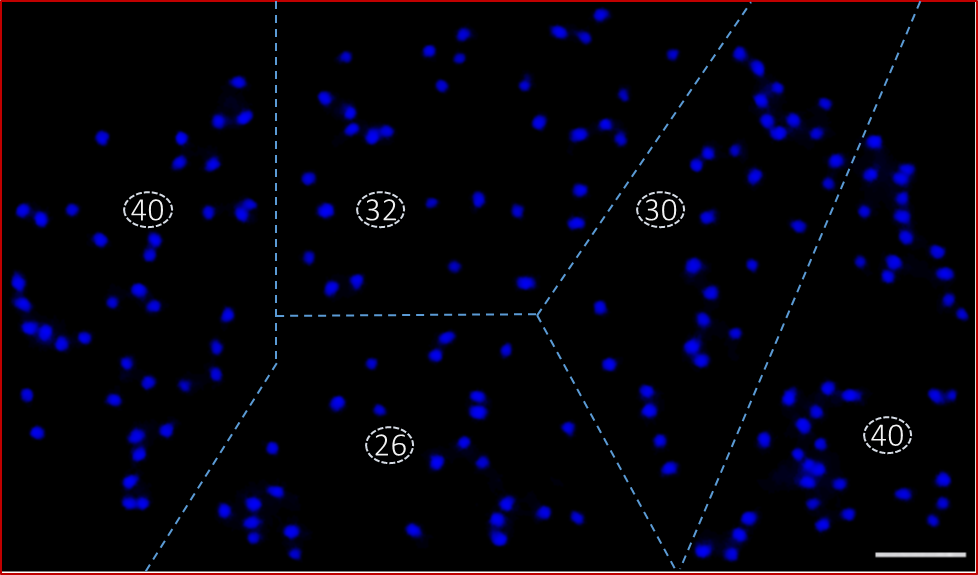


**Figure S2a.** Somatic root tip chromosome spread (late prophase/prometaphase) of *Adansonia digitata* L. stained with DAPI. The spread is divided into five partitions to faciltate counting chromosomes (40 + 32 + 30 + 26 + 40 = 168). Scale bar is 5 µm.

**Fig. S2b. DAPI Stained Chromosome Spread for Chromosome Counting in *Adansonia digitata***


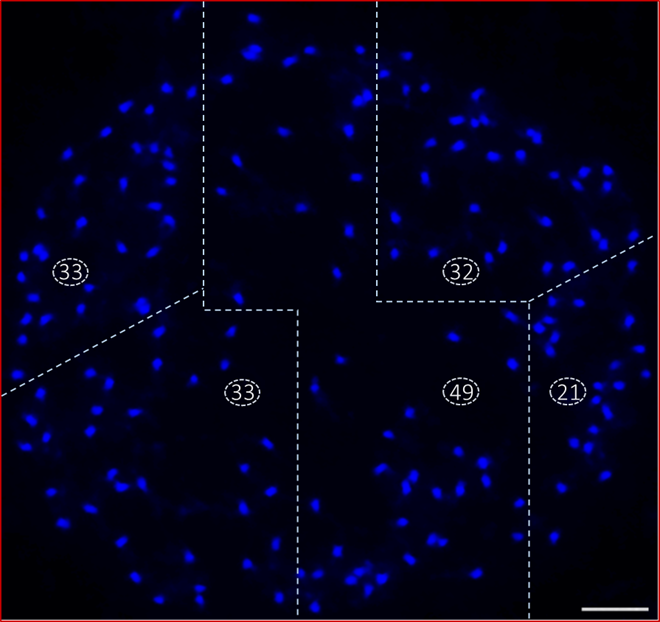


**Figure S2b.** Somatic root tip chromosome spread (late prophase) of *Adansonia digitata* L. stained with DAPI. The spread is divided into five partitions to faciltate counting chromosomes (33 + 32 + 33 + 49 + 21 = 168). Scale bar is 5 µm.

**Fig. S2c. DAPI Stained Chromosome Spread for Chromosome Counting in *Adansonia digitata***


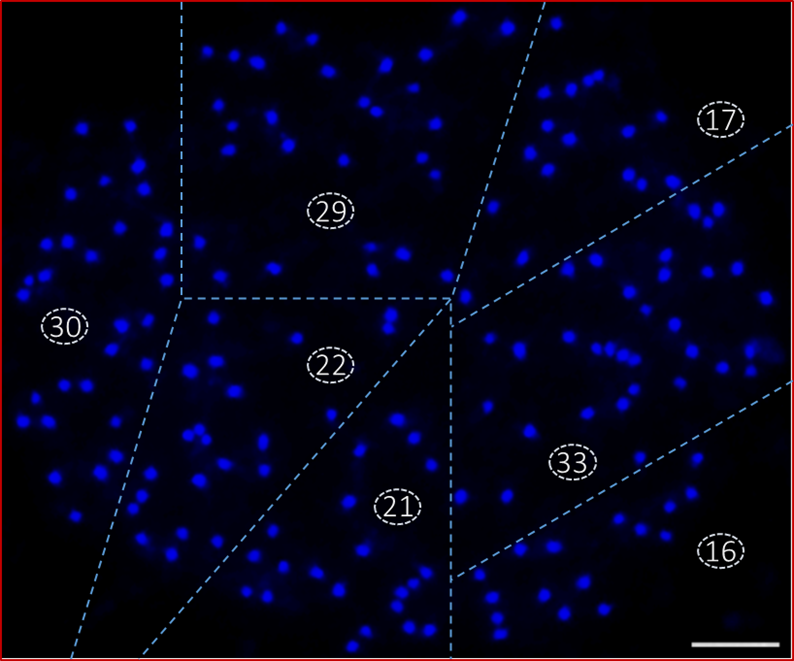


**Figure S2c.** Somatic root tip chromosome spread (late prophase/prometaphase) of *Adansonia digitata* L. stained with DAPI. The spread is divided into seven partitions to faciltate counting chromosomes (30 + 29 + 17 + 22 + 21 + 33 + 16 = 168). Scale bar is 5 µm.

**Fig. S2d. DAPI Stained Chromosome Spread for Chromosome Counting in *Adansonia digitata***

**
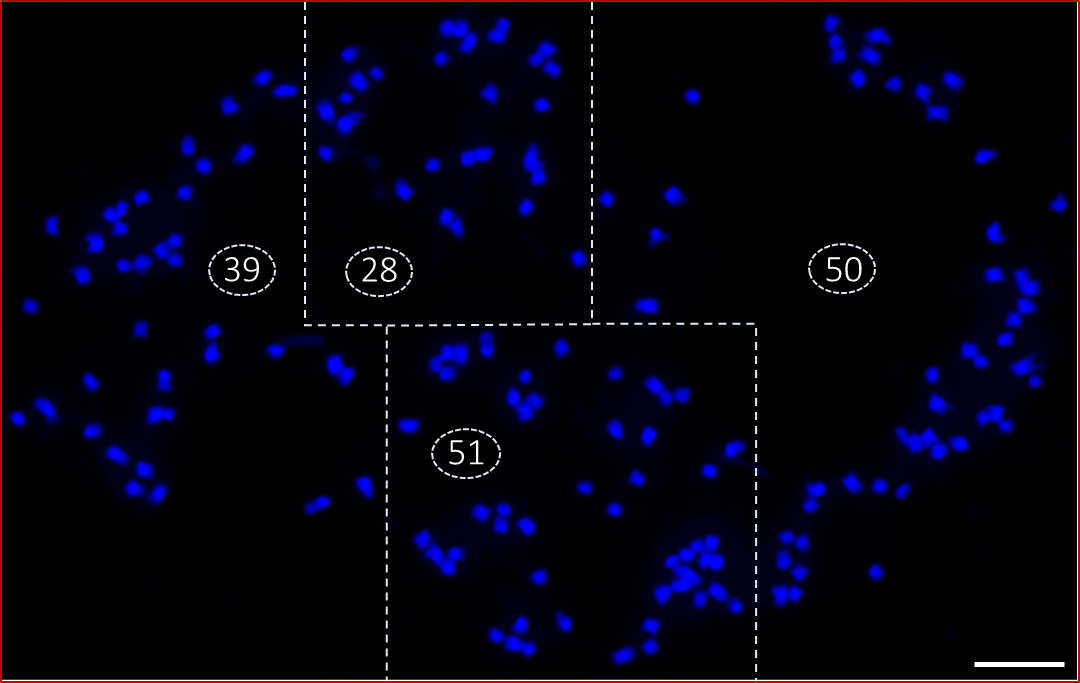
**

**Figure S2d.** Somatic root tip chromosome spread (metaphase) of *Adansonia digitata* L. stained with DAPI (same cell as in Fig. 3, enlarged image). The spread is divided into four partitions to faciltate counting chromosomes (39 + 28 + 50 + 51 = 168). Scale bar is 5 µm.

**Fig. 2Se. DAPI Stained Chromosome Spread for Chromosome Counting in *Adansonia digitata
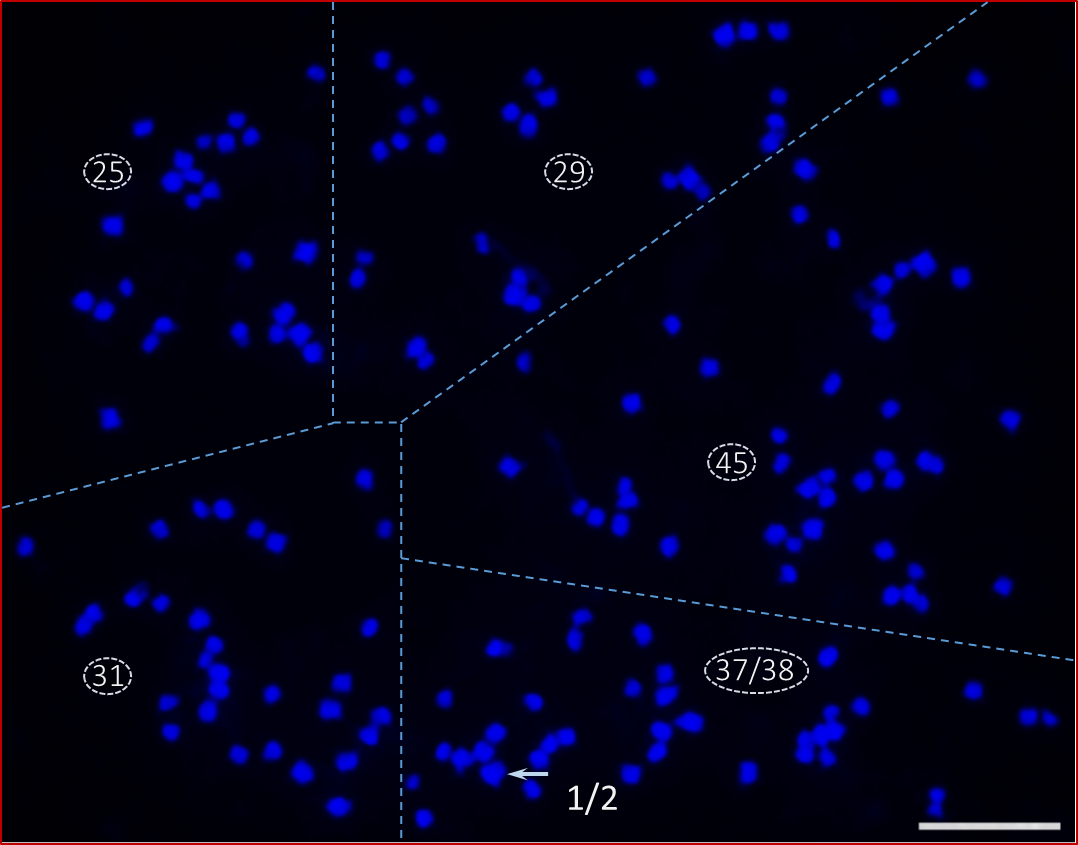
***

**Figure S2e.** Somatic root tip chromosome spread (prometaphase) of *Adansonia digitata* L. stained with DAPI. The spread is divided into five partitions to faciltate counting chromosomes (25 + 29 + 45 + 31 + 37/38 = 167/168). Two chromosomes are overlapping (arrow) which could result in an erroneous count of 167 chromosomes instead of the correct number, 168. Scale bar is 5 µm.

**Fig. 2f. DAPI Stained Shromosome Spread Not Considered for Chromosome Counting in *Adansonia digitata***


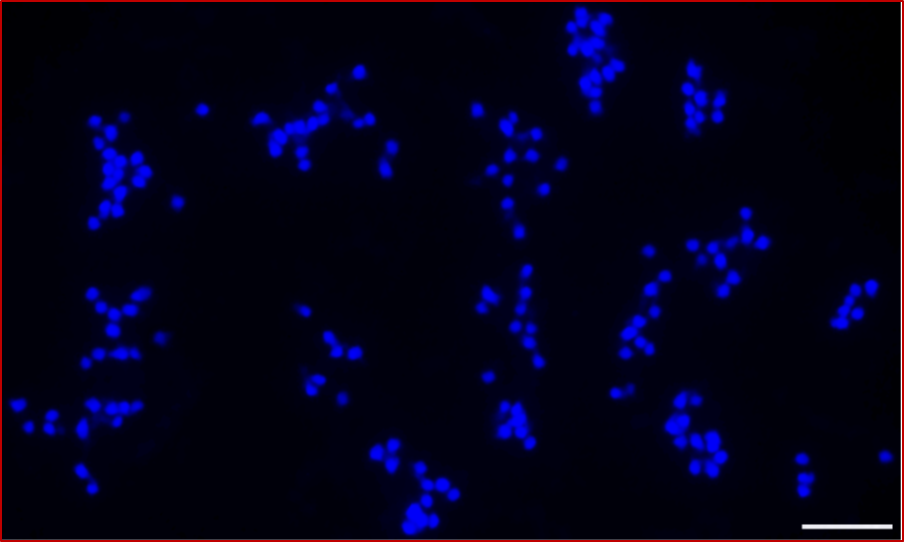


**Figure S2f.** Somatic root tip chromosome spread (late prophase) of *Adansonia digitata* L. stained with DAPI. We would not consider a spread like this one for chromosome counting because of overlapping chromosomes. Scale bar is 5 µm.

**Fig. S2g. DAPI Stained Chromosome Spread Not Considered for Chromosome Counting in *Adansonia digitata***


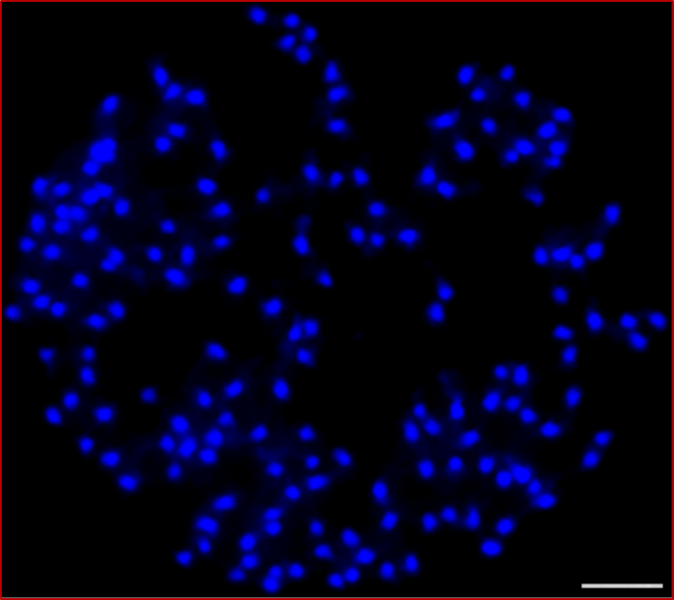


**Figure S2g.** Somatic root tip chromosome spread (late prophase) of *Adansonia digitata* L. stained with DAPI. We would not consider a spread like this one for chromosome counting because chromosomes are still most likely encircled by nuclear envelops and some chromosomes are clumped. Scale bar is 5 µm.

**Fig. S2h. DAPI Stained Metaphase Spread Not Considered for Chromosome Counting in *Adansonia digitata***


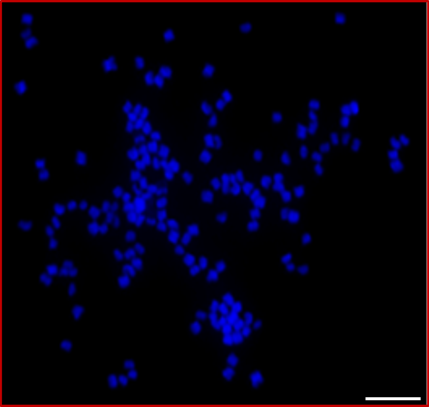


**Figure S2h.** Somatic root tip chromosome spread (metaphase) of *Adansonia digitata* L. stained with DAPI. We would not consider a spread like this one for chromosome counting because chromosomes are not clearly separated. Scale bar is 5 µm.

**Fig. S3. First FISH with 45S and 5S rDNA Oligo Probes and Second FISH with 45S rDNA and Telomere Oligo Probes in *Adansonia digitata***


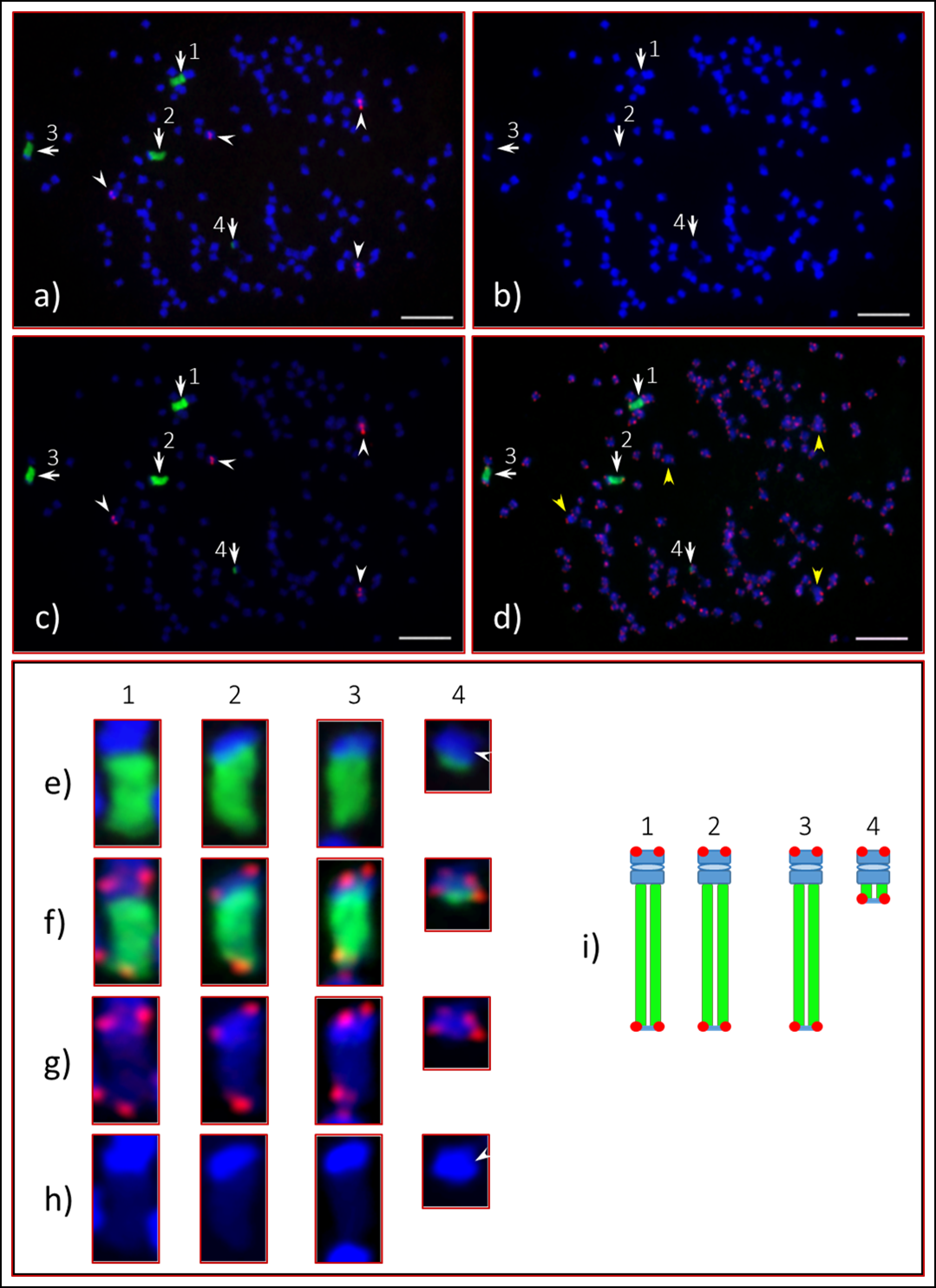


**Figure S3.** A somatic (root tip) metaphase chromosome spread of of *Adansonia digitata* L. analyszed twice with FISH using 45S and 5S rDNA oligo probes (first FISH) and 45S rDNA and ATRS oligo probes (second FISH). a) Three massive and one small 45S rDNA green FISH signals (arrows, chromosomes are numbered as 1, 2, 3 and 4) and four 5S rDNA red signals (arrowheads) observed. b) Lightly shaded nucleolus organizer (NOR) areas observed from the large 45S rDNA bearing chromosomes (arrows, 1, 2 and 3); c) Same spread with with reduced DAPI; the green and red signals are from 45S and 5S rDNA probes are clearly visible;d) Same spread, second FISH with 45S rDNA as a control (green signals, arrows) and telomere (red signals) oligo probes after washing off the probes from the first FISH, there is no sign of 5S rDNA signals (yellow arrowheads). e) Enlarged images of the 45S rDNA (green signal) bearing chromosomes. (f) Signals from 45S rDNA (green) and telomere (red) probes. g) Chromosomes with telomere signals. h) Lightly shaded DAPI can be observed in each of the 45S rDNA chromosomes, arrowhead pointed at the centromeric region of the fourth chromosome. i) Diagrammatic representation of the four 45S rDNA bearing chromosomes. Scale bar is 5 µm.

**Fig. S4. FISH with 45S and 5S rDNA Oligo Probes on Interphase Nucleus and a Diagrammatic Model of 45S rDNA Distribution in *Adansonia digitata***


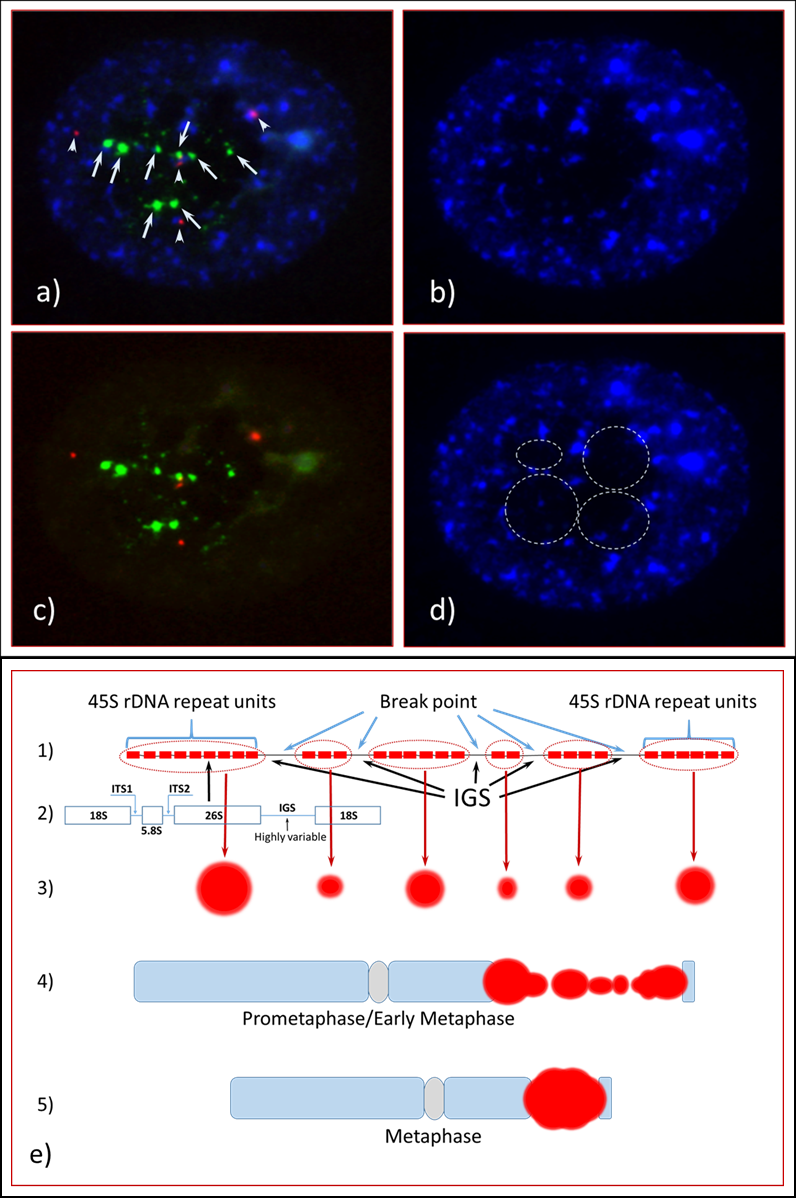


**Figure S4.** Interphase nucleus with 45S and 5S rDNA FISH signals on chromosomes of *Adansonia digitata*and a diagrammatic model of 45S rDNA in interphase, pro-metaphase and metaphase cells. a) Numerous 45S rDNA FISH signals including eight major bodies (green, arrows) and four 5S rDNA signals (red, arrowheads) are observed in DAPI stained nucleus. b) Same interphase nucleus stained with DAPI; brightly stained bodies are from heterochromatin blocks and lightly stained bodies are from euchromatin blocks; the center of the nucleus appears to be hollow. c) Same nucleus with no DAPI; the clearly visible green and red signals are from 45S and 5S rDNA probes, respectively. d) The encircled dotted lines are nucleoli. e) Structure of 45S rDNA and progression of a cell from interphase to metaphase. 1) 45S rDNA units on nucleolus organizer region (NOR) in interphase nucleus. 2) 45S rDNA unit composed of 18S-ITS1-5.8S-ITS2-26S, and an IGS is connected to two 45S rDNA units; there are six clusters  of 45S rDNA repeat units forming various sizes of signal (small to large) depending on the number of 45S rDNA repeat units in each cluster. These clusters get separated during chromosome preparation thus resulting in a multiple FISH signals (various intensities) on the spreads. 3) An interphase nucleus with six different sizes (circles) of 45S rDNA signals (red). 4) A prometaphase chromosome with a number of concentrated, but dispersed 45S rDNA signals. 5) A metaphase chromosome with one aggregated, large signal.

**Fig. S5. FISH of Mid-Prophase Chromosome Spread with 45S and 5S rDNA Oligo Probes in *Adansonia digitata***


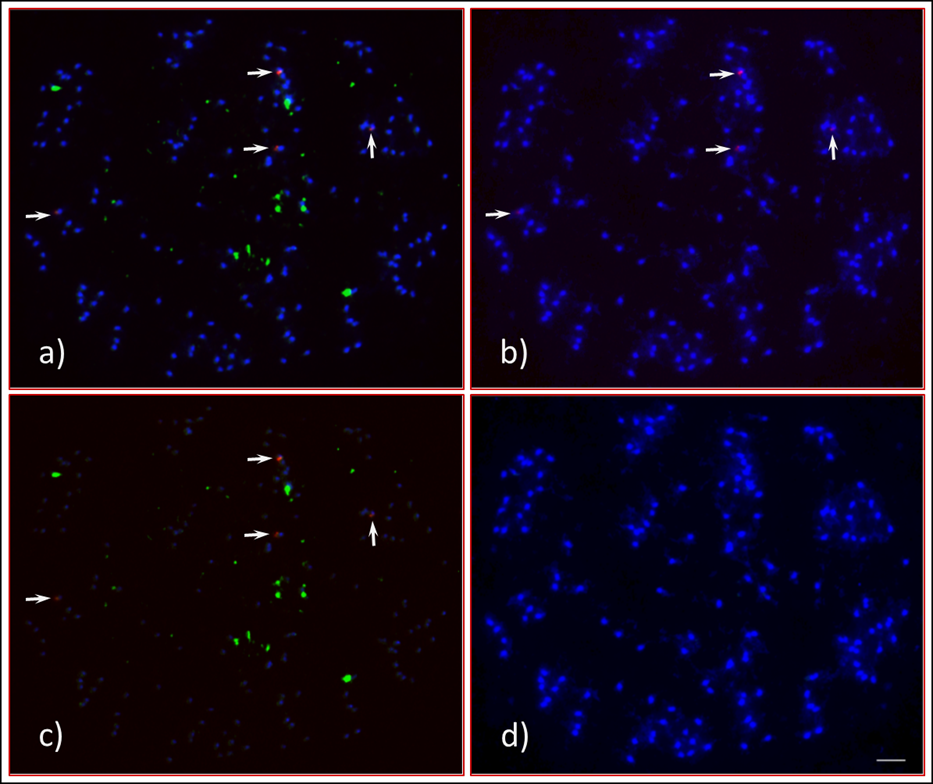


**Figure S5. FISH of** mid-prophase chromosome spread of *Adansonia digitata* L. with 45S (green signals) and 5S (red signals) rDNA oligo probes; about 20 45S rDNA FISH signals sporadically distributed in the cell (a and c). b) Four 5S rDNA signals (arrows) can be observed in DAPI-stained chromosomes. c) Same cell with reduced DAPI to brighten the green (45S rDNA signals) and red (5S rDNA signals, arrows). d) DAPI stained chromosomes. Scale bar is 5 µm.

**Fig. S6. FISH of Mid-Prophase Chromosome Spread with 45S and 5S rDNA Oligo Probes in *Adansonia digitata***


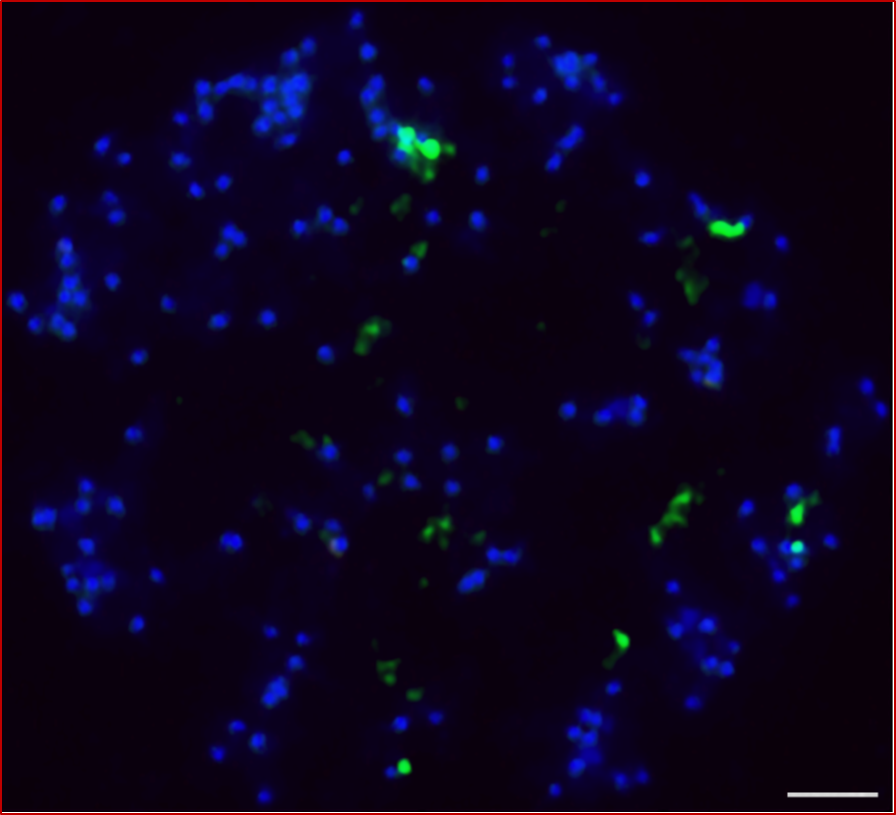


**Figure S6.** FISH of a mid-prophase chromosome spread of *Adansonia digitata* L. showing more than 35 45S rDNA FISH signals (green) sporadically distributed in the cell with DAPI stained chromosomes. Scale bar is 5 µm.

**Fig. S7. FISH with Telomere and 45S rDNA Oligo Probes in Interphase and Prophase Cells in *Adansonia digitata***


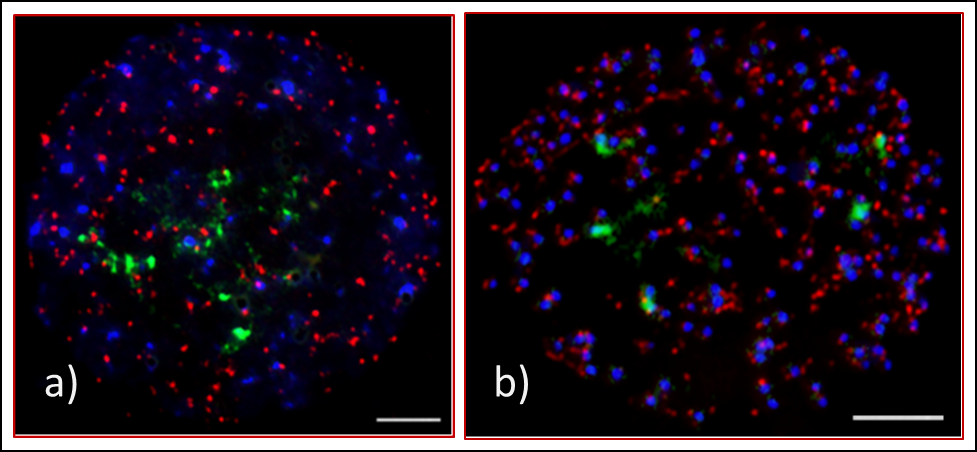


**Figure S7.** FISH of interphase and prophase cells of *Adansonia digitata* L. with 45S rDNA and telomere oligo probes. a) Numerous (about 50+) 45S rDNA FISH signals (green) observed in an interphase nucleus. b) About 10 scattered (medium to major) signals and five large bodies of 45S rDNA signals observed in a late prophase cell. Abundant telomere signals (red) observed throughout the interphase nucleus (a) and prophase cell (b). Scale bars are 5 um.

**Fig. S8.** *Adansonia digitata* L. Seedlings


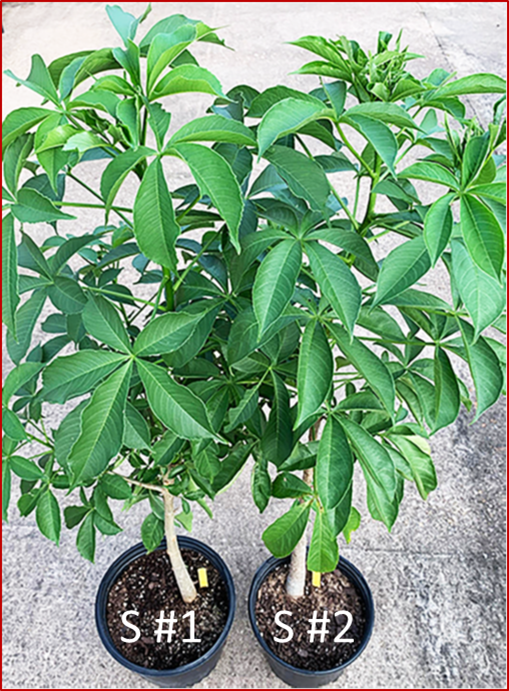


**Figure S8.** *Adansonia digitata* L. seedlings, S#1 (Seedling-1) and S#2 (Seedling-2) used for cytological analyses.
